# *AnACor2.0*: a GPU-accelerated open-source software package for analytical absorption corrections in X-ray crystallography

**DOI:** 10.1107/S1600576724009506

**Published:** 2024-11-04

**Authors:** Yishun Lu, Karel Adámek, Tihana Stefanic, Ramona Duman, Armin Wagner, Wesley Armour

**Affiliations:** a Oxford e-Research Centre, Department of Engineering Science, University of Oxford, 7 Keble Road, OxfordOX1 3QG, United Kingdom; b Diamond Light Source Harwell Science & Innovation Campus DidcotOX11 0DE United Kingdom; DESY, Hamburg, Germany

**Keywords:** absorption correction, ray-tracing, long-wavelength crystallography, CUDA acceleration, software

## Abstract

Analytical absorption corrections are employed in scaling diffraction data for highly absorbing samples, such as those used in long-wavelength crystallography, where empirical corrections pose a challenge. *AnACor2.0* is an accelerated software package developed to calculate analytical absorption corrections. It accomplishes this by ray-tracing the paths of diffracted X-rays through a voxelized 3D model of the sample. Due to the computationally intensive nature of ray-tracing, the calculation of analytical absorption corrections for a given sample can be time consuming. Three experimental datasets (insulin at λ = 3.10 Å, thermolysin at λ = 3.53 Å and thaumatin at λ = 4.13 Å) were processed to investigate the effectiveness of the accelerated methods in *AnACor2.0*. These methods demonstrated a maximum reduction in execution time of up to 175× compared with previous methods. As a result, the absorption factor calculation for the insulin dataset can now be completed in less than 10 s. These acceleration methods combine sampling, which evaluates subsets of crystal voxels, with modifications to standard ray-tracing. The bisection method is used to find path lengths, reducing the complexity from *O*(*n*) to *O*(log_2_ *n*). The gridding method involves calculating a regular grid of diffraction paths and using interpolation to find an absorption correction for a specific reflection. Additionally, optimized and specifically designed CUDA implementations for NVIDIA GPUs are utilized to enhance performance. Evaluation of these methods using simulated and real datasets demonstrates that systematic sampling of the 3D model provides consistently accurate results with minimal variance across different sampling ratios. The mean difference of absorption factors from the full calculation (without sampling) is at most 2%. Additionally, the anomalous peak heights of sulfur atoms in the Fourier map show a mean difference of only 1% compared with the full calculation. This research refines and accelerates the process of analytical absorption corrections, introducing innovative sampling and computational techniques that significantly enhance efficiency while maintaining accurate results.

## Abstract 2

*AnACor2.0* significantly accelerates the calculation of analytical absorption corrections in long-wavelength crystallography, achieving up to 175× speed improvements. This enhancement is achieved through innovative sampling techniques, bisection and gridding methods, and optimized CUDA implementations, ensuring efficient and accurate results.

## 1. Introduction

Processing X-ray crystallography diffraction data involves integrating diffraction images to extract reflection intensities. Integrated intensities, which are influenced by experimental conditions, require data scaling to reflect the true structure-factor amplitudes, 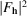. This involves correcting for systematic effects such as sample illumination, variations in beam intensity, radiation damage and absorption effects. By fitting a scaling model that accounts for these factors, an internally consistent dataset can be produced, improving the quality of merged symmetry-equivalent reflections.

The absorption effect is primarily determined by the crystal’s composition, its shape and the wavelength of the X-ray beam. In macromolecular crystallography, various data reduction software packages, such as *AIMLESS* (Evans & Murshudov, 2013 ▸), *hkl3000* (Minor *et al.*, 2006 ▸), *SADABS* (Bruker, 2016 ▸) and *DIALS* (Winter *et al.*, 2018 ▸), employ spherical harmonics corrections to address absorption effects. This method typically relies on data multiplicity, which is unaffected by the sample’s material and geometry. However, the effectiveness of this approach diminishes when it is difficult to obtain multiple datasets from different goniometer orientations, such as with radiation-sensitive crystals in low-symmetry space groups. In such cases, the limited number of symmetry-equivalent reflections hampers the success of spherical harmonics correction. Introducing analytical absorption correction improves data-scaling quality in these scenarios because it does not rely on data multiplicity. *AnACor1.0* (Lu *et al.*, 2024 ▸) applies this method, which is based on equation (1) below and utilizes a 3D model of the sample,
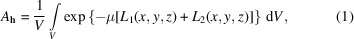
where *L*_1_(*x*, *y*, *z*) and *L*_2_(*x*, *y*, *z*) (hereafter referred to as *L*_1_ and *L*_2_) are the incident and diffracted X-ray path lengths to and from each crystal element d*V*, μ is the absorption coefficient of the crystal, and *A*_**h**_ is the inverse absorption factor (referred to as the absorption factor in the following context) (Albrecht, 1939 ▸). For the implementation of analytical absorption correction on a voxelized 3D model, the integral in equation (1) can be reformulated discretely (Lu *et al.*, 2024 ▸):
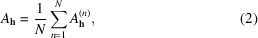
where *N* is the number of crystal voxels in the 3D model exposed to the X-ray beam. This is because the crystal volume (Angel, 2004 ▸) is the only component that contributes to the X-ray diffraction. In a crystallographic experiment, it is common for the sample to consist of multiple materials. As a result, the determination of the absorption correction factor 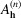 for a crystal voxel can be reformulated as follows: 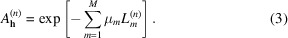
The symbol 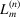 denotes the combined length of the incident length 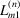 and the diffracted length 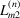 as they pass through the material *m* being diffracted at the crystal voxel *n*.

In *AnACor1.0*, the path lengths are determined by a ray-tracing method (described in Section 2.2), which needs to traverse a large number of voxels in the 3D model to obtain accurate results. Macromolecular crystallography often requires examination of thousands of reflections, with the number of crystal voxels in the 3D model *N* reaching into the millions. This makes processing all reflections and voxels a significant computational obstacle for efficient absorption correction. In the previous work (Lu *et al.*, 2024 ▸), *AnACor1.0* performed analytical absorption correction by Python and *Numba 0.56.2* (Lam *et al.*, 2015 ▸) to enhance computational efficiency. It employed a systematic sampling method with a 0.05% sampling ratio, reducing the processing time for a dataset to approximately 40 min. However, this is still too slow for quickly analysing many datasets, especially for large samples with numerous crystal voxels. To manage this load, we present *AnACor2.0*, an innovative software solution designed to streamline analytical absorption correction by incorporating new, sophisticated computational strategies

*AnACor2.0* employs sampling methods to process fewer crystal voxels *N* while maintaining result accuracy. It also uses a bisection approach to enhance the standard ray-tracing method for determining path lengths. Instead of traversing every voxel along the diffracting ray, the bisection method iteratively identifies the middle voxel’s material to locate all material boundaries, which are used to calculate the path lengths. This significantly improves time complexity, which is crucial for large samples with many voxels. Additionally, *AnACor2.0* includes a module that calculates absorption gridding maps for each crystal voxel *n* and uses interpolation techniques to determine the path length for a given direction of the diffracting ray. This approach reduces repetitive computation of diffracting rays from similar directions. One of the key characteristic modules of *AnACor2.0* is its utilization of NVIDIA’s CUDA platform for acceleration. This module utilizes the capabilities of parallel computing on GPUs, a computational accelerator that enables concurrent calculations across many processing units. Through these approaches, *AnACor2.0* can significantly reduce computing times.

This study explores three standout experimental datasets: insulin at λ = 3.10 Å, thermolysin at λ = 3.53 Å and thaumatin at λ = 4.13 Å, all in high-symmetry space groups. Insulin is spherical, thaumatin is pyramidal and thermolysin is asymmetrical, demonstrating that *AnACor2.0* is versatile and applicable to various sample shapes.

This study assesses the performance of different sampling methods and acceleration techniques on analytical absorption correction in these experiments. It employs analytical absorption correction followed by spherical harmonics correction (ACSH) (Lu *et al.*, 2024 ▸) for data scaling, comparing absolute differences in absorption factors and analysing relative variations in anomalous peak heights in the anomalous difference Fourier maps of the crystals. Further details on merging statistics and anomalous peak heights for various absorption correction strategies [no correction, spherical harmonics correction (Beilsten-Edmands *et al.*, 2020 ▸), analytical absorption correction (Lu *et al.*, 2024 ▸) and ACSH] are provided in the supporting information.

All results were obtained using the Oxford Advanced Research Computing supercomputer (Richards, 2015 ▸), on a single node with an Intel Xeon Platinum 8268 CPU with 48 cores. We evaluated GPU performance on NVIDIA V100, A100 and H100 GPUs. Section 3 compares the computational time of the acceleration methods with the original baseline presented in *AnACor1.0* (Lu *et al.*, 2024 ▸).

*AnACor2.0* is publicly released at https://github.com/yishunlu-222/AnACor2.0.git with a GNU General Public Licence v3.0.

## 2. Methodology

### 2.1. Data preparation and implementation

In selecting the protein samples for this study, we aimed for morphological variation and to obtain crystals in high-symmetry space groups, since previously we had focused only on low-symmetry crystals.

All crystallization experiments were performed using the sitting-drop vapour-diffusion method at 20°C in Swissci (UVXPO-2 lens) 96-well plates, by mixing 100 nl of protein solution with 100 nl of crystallization buffer. Crystals of thaumatin (Sigma, T7638) were obtained from a 50 mg ml^−1^ solution of the protein powder suspended in deionized water and a crystallization buffer consisting of 100 m*M* ADA pH 6.5, 750 m*M* potassium sodium tartrate, dissolved in saturated 5,5′-dithiobis(2-nitrobenzoic acid) water, and 25% glycerol. The crystal used in this study had dimensions of 110 × 84 × 75 µm^3^ in size. Insulin powder (Sigma, I5500) was dissolved to a concentration of 25 mg ml^−1^ in 50 m*M* Na_2_HPO_4_ pH 10.5 and 10 m*M* ethylenediaminetetraacetic acid, and crystallized by mixing with 20% ethylene glycol. The crystal used here had dimensions of 35 × 45 × 45 µm^3^ in size. To crystallize thermolysin, the protein powder (Sigma, P1512) was dissolved in a buffer consisting of 50 m*M* 2-(*N*-morpholino)ethanesulfonic acid pH 6.0, 45% dimethyl sulfoxide and 50 m*M* sodium chloride, to a concentration of 50 mg ml^−1^, and mixed with 1.2 *M* ammonium sulfate. The thermolysin crystal selected for this study measured 230 × 70 × 70 µm^3^ in size.

Sample preparation for in-vacuum X-ray crystallography followed a previously published procedure (Duman *et al.*, 2021 ▸). Data were collected on the long-wavelength beamline I23 at Diamond Light Source (Wagner *et al.*, 2016 ▸), with tomography data collection performed at the same wavelength, immediately following the diffraction experiment, as previously described (Lu *et al.*, 2024 ▸). All tomography datasets were collected at 100% transmission and the beam adjusted to a size of 700 × 700 µm^2^. For thaumatin, 360° of diffraction data were collected at a wavelength of λ = 4.13 Å, with a top-hat beam size of 200 × 200 µm^2^, 50% transmission and a 0.1 s per 0.1° exposure. The tomography dataset consisted of 1800 projections, 20 dark images (no X-ray beam on the sample) and 20 flat-field images (sample out of the beam), recorded with an exposure of 0.2 s per 0.1°. The insulin diffraction data were measured at λ = 3.1 Å as a 360° sweep, with 50% transmission, a beam size of 100 × 100 µm^2^ and an exposure of 0.1 s per 0.1°. For tomography, 1800 projections, 20 dark images and 20 flat-field images were collected with an exposure of 0.1 s per 0.1°. For the thermolysin diffraction data, measured at λ = 3.54 Å, 360° of data were collected with a beam size of 350 × 350 µm^2^, 15% transmission and 0.1 s per 0.1° exposure, using a kappa goniometer setting of −70° to ensure data completeness, since the rod-like crystal was aligned with the rotation axis. The tomography dataset consisted of 900 projections, 20 dark images and 20 flat-field images, recorded with an exposure of 0.2 s per 0.2°. The diffraction data were indexed and integrated with *DIALS* (Winter *et al.*, 2018 ▸).

We use segmented tomographic reconstructions as our 3D models of the samples. The tomography data were processed with the *SAVU* pipeline (Kazantsev *et al.*, 2022 ▸), using standard flat-field correction, followed by ring artefact removal (Vo *et al.*, 2018 ▸) and reconstruction by filter-back projection with *TomoPy* (Gürsoy *et al.*, 2014 ▸). The reconstruction datasets were cropped from an initial size of 1600 × 200 × 1200 voxels to remove unnecessary background and reduce the size of the dataset. The final dimensions of the tomographic datasets were 1120 × 1001 × 1001 voxels for thaumatin, 470 × 1000 × 1000 voxels for insulin and 1210 × 1001 × 1001 voxels for thermolysin, with a voxel size of 0.3 × 0.3 × 0.3 µm^3^. The reconstruction images were subsequently annotated using the segmentation software *Avizo* (Thermo Fisher), resulting in every pixel being labelled as one of the three materials present in the sample: crystal, solvent, loop or, alternatively, background.

To calculate the absorption correction factor, we use a segmented 3D model in an array data structure, absorption coefficients, and a table of directional vectors for the incident and diffracted X-rays corresponding to the reflections **h**. The absorption coefficients can be determined as described by Lu *et al.* (2024 ▸) or provided as input. The absorption coefficients of insulin, thermolysin and thaumatin are presented in Table 1 ▸. Unlike *AnACor1.0*, which used Python, *AnACor2.0*’s core computational modules are implemented as C function calls with CPU parallelism via OpenMP. These modules also have a Python interface and can be called directly from Python using ctypes. The output is a collection of analytical absorption factors *A*_**h**_ in JSON format, arranged by the order of reflections in the input table. Once the calculations are complete, the analytical absorption correction is applied in the data-scaling process using dials.scale in *DIALS* (Winter *et al.*, 2018 ▸; Beilsten-Edmands *et al.*, 2020 ▸) with the flag of analytical_correction = True.

The absorption factors obtained through the standard ray-tracing technique with no sampling are established as the benchmarks for each dataset. The use of mean absolute percentage differences between the absorption factors of acceleration methods and a no-sampling standard method helps to assess the performance differences in acceleration. The differences are calculated as [abs(*A*_acc_ − *A*_no_)] /*A*_no_ on an absolute scale, where *A*_acc_ and *A*_no_ are, respectively, the absorption factors of the same reflection in the accelerated method and the no-sampling standard method. We also considered the peak heights in the anomalous difference Fourier maps of experimental datasets to examine if the final data quality prevails after applying acceleration methods. The published structures, Protein Data Bank (PDB) ID 4a7e (Burkhardt *et al.*, 2012 ▸) for insulin, PDB ID 1kei for thermolysin and PDB ID 1rqw for thaumatin, are used as starting models for the *Dimple* pipeline (https://ccp4.github.io/dimple/). The --anode option (Thorn & Sheldrick, 2011 ▸) is used to calculate anomalous difference Fourier maps with anomalous peak heights, and the option --free-r-flags in the *Refmac* refinement (Murshudov *et al.*, 1997 ▸) step ensures the same R-free flags for all acceleration strategies. The peak heights of sulfur atoms in insulin, thermolysin and thaumatin are selected for comparison with no-sampling results. Similar to the absorption factors, the percentage differences of peak heights are calculated as [abs(*H*_acc_ − *H*_no_)] /*H*_no_, where *H*_acc_ and *H*_no_ are the anomalous peak heights of the same atoms in the accelerated method and no-sampling standard method, respectively.

### 2.2. Standard ray-tracing method

The standard ray-tracing approach assumes X-rays incident upon a crystal voxel *n*, subsequently undergoing diffraction at that voxel, and consists of two algorithms: traversal and length calculation. During each ray traversal along the incident and scattered X-ray directions, the voxels’ coordinates and their related material labels are calculated and subsequently recorded. After finishing the traversal, the absorption factors can be calculated from the recorded information via the length calculation algorithm.

**Figure 1:**
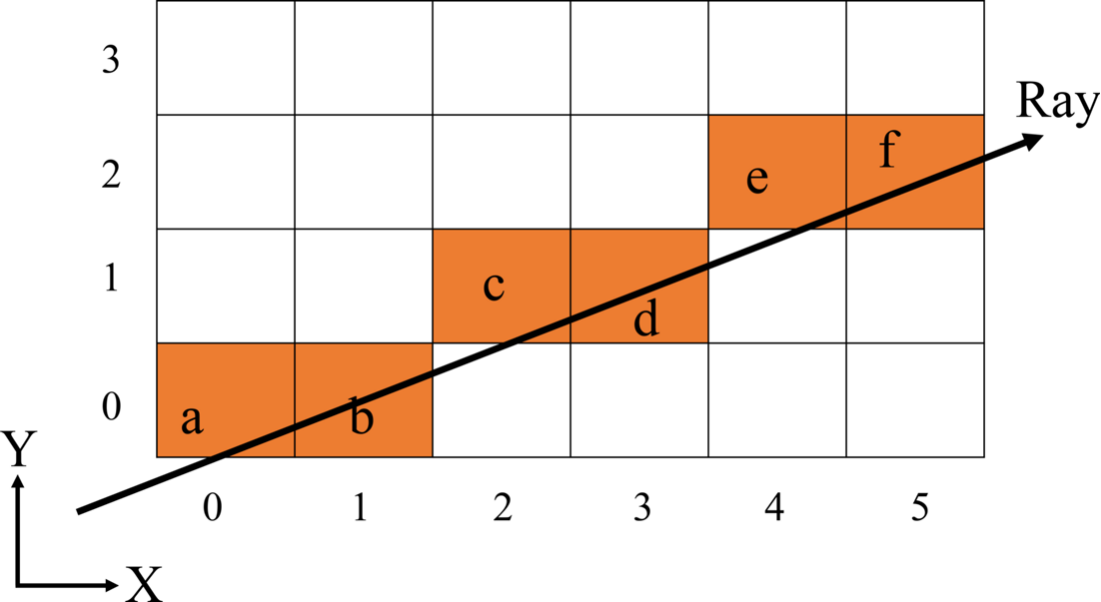
Schematic diagram of ray-tracing traversal algorithm with a ray traversing from bottom left to top right.

The construction of the model involves stacking 2D segmented slices of the tomographic reconstruction, resulting in a 3D array that can be referred to as a cuboid with six planes. The traversal algorithm is inspired by the fast voxel traversal algorithm (Amanatides & Woo, 1987 ▸), which is used in voxel traversal through a 3D array. We consider a 2D case to better illustrate the traversal algorithm in Fig. 1 ▸, and it is easy to extend to 3D. A ray passes the pixels from bottom left to top right, with an equation of 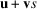, where **v** is the direction of the ray and **u** is a point on the ray. In Fig. 1 ▸, the ray splits down into intervals of *s* along the *X* axis, corresponding to one pixel. Then, all of the coordinates of the traversed pixels *b*, *c*, *d*, *e* and *f* can be calculated by starting at pixel *a* [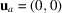] in the ascending interval order (*s* = 1, 2, 3…*n*, where *n* is the maximum interval and *n* = 5 in Fig. 1 ▸), and then rounding to the nearest pixel coordinates. This is because, to calculate the path length, the start point and the end point are crucial and the intermediate pixels along the ray are less significant, where only one pixel is enough for each interval. That is why the pixel below pixel *e* does not count. Hence, in this case, the path length from *a* to *f* is the Euclidean length between coordinates (0, 0) and (5, 2).

The determination of which axis should split depends on the plane of the array that intersects with the ray. This ensures that all calculated results fall within the array’s range, and every pixel along the ray can be accurately identified. For instance, if the *Y* axis is chosen to split in Fig. 1 ▸, the interval sequence would be *s* = 1, 2, 3. Consequently, the calculated coordinates of *b* would be the same as the coordinates of point *a*, and this pattern would continue through the coordinates of (*c*, *d*) and (*e*, *f*). Therefore, the path length calculations are incorrect.

More specifically, there are two stages in the traversal algorithm: initialization and incremental traversal. The first phase determines the exit points of the ray (such as pixel *f* in Fig. 1 ▸), which can confirm which axis to split down and if this interval order is ascending or descending (*s* = 1, 2, 3…*n* or *s* = *n*…3, 2, 1). This is in contrast to the fast voxel traversal algorithm (Amanatides & Woo, 1987 ▸), where our algorithm only considers incrementing or decrementing along one direction (either *x* or *y*). In the incremental traversal stage, in Fig. 1 ▸, the coordinates of the new pixel are computed by Δ*Y* and Δ*X* along the *y* and *x* axes from the starting pixel (*X*, *Y*) with rounding to the nearest integers. Δ*X* indicates how far along the ray we must move along the *x* axis component in one step *s*. It is straightforward to see that Δ*Y* is determined by multiplying the direction of the ray **v**. The basic loop of Fig. 1 ▸ is outlined in Algorithm 1:
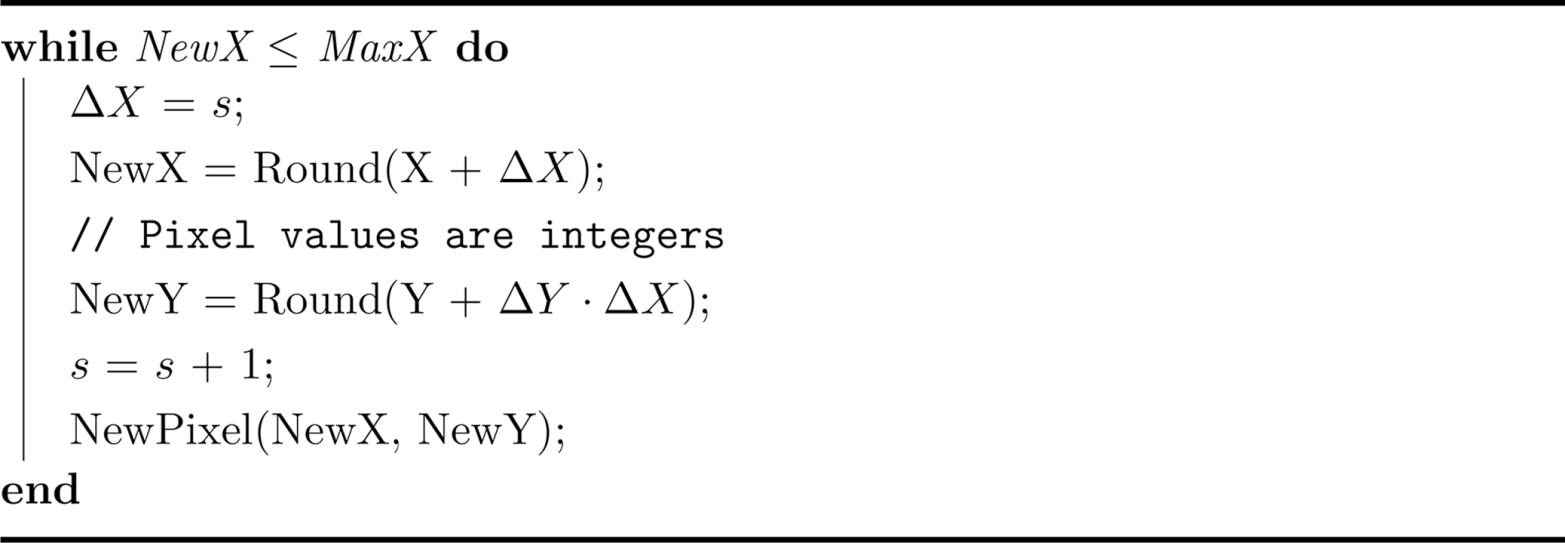


In 3D cases, the initialization in the traversal algorithm that aims to find the exit faces of the ray becomes more difficult. A ray-casting technique is used, as outlined in equations (4)–(6): 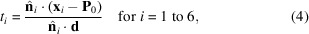

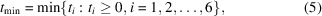

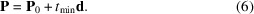
The cuboid model consists of six faces, which can be mathematically represented as six planes that extend to infinity. These planes are defined by the vertices of the cuboid within the vector space. The calculation in equation (4) determines the distance *t*_*i*_ between the crystal coordinate and the intersection with the plane. This is done by utilizing the unit normal vector 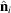 of the plane, the vertex coordinates **x**_*i*_ on the plane, the directional vector **d** of the X-ray beam and the crystal point **P**_0_. The vector **d** intersects with points on all six faces within the infinite vector space. The minimal value of the non-negative *t*_*i*_ represents the location where plane *i* connects with the cuboid in the positive direction of the vector **d**. Overall, the exit coordinates can also be determined by equation (6) and this identifies the specific face of the cuboid that intersects with the X-ray beam, which helps finish initialization.

**Figure 2:**
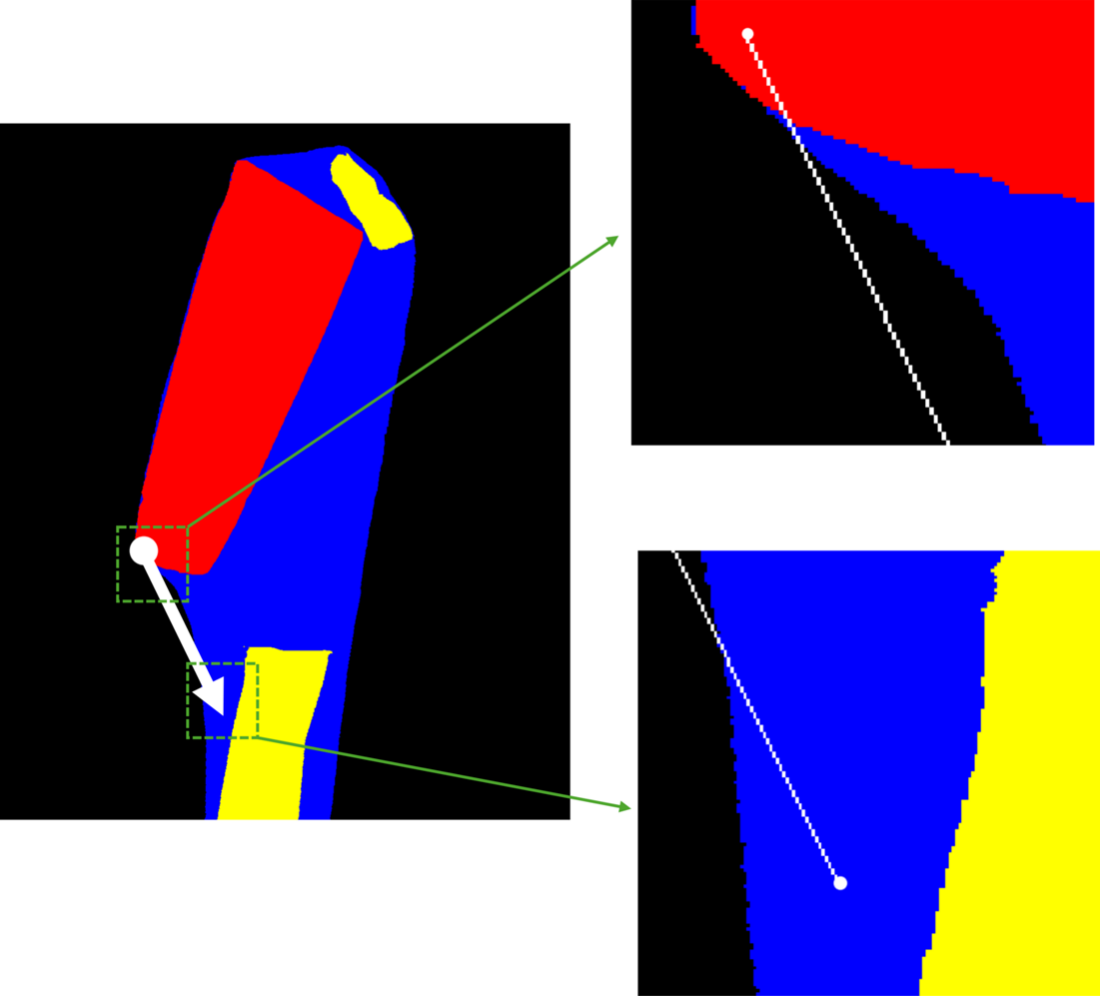
A ray-tracing path marked in white for a tomographic reconstruction slice of thermolysin (black, vacuum; red, crystal; yellow, loop; blue, mother liquor). Two subplots in the green dashed regions demonstrate the zigzag patterns where all the pixels on the ray-tracing path are marked in white. The diffracted path contains a large region of vacuum/air.

In the path length calculation algorithm for incident path length 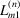, the crystal voxel *n* is considered as the starting point for the X-ray traversal rather than the X-ray source, so the direction of the incoming vector is reversed and it originates from the crystal voxel *n*. The algorithm iterates until it encounters the boundary of the 3D model and the coordinates and the corresponding labelled materials are recorded during the traversal. When the iteration stops, the total path length is calculated as the Euclidean distance between the starting point and the last voxel where the iteration stops. As depicted in Fig. 2 ▸, the coordinates recorded exhibit a zigzag pattern along the X-ray path due to the voxelization process. To mitigate this zigzag effect, the individual path lengths [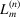] for material *m* are determined by first calculating the proportion of the total path length that the material *m* occupies during traversal and then multiplying this proportion by the total path length. This product is combined with the voxel size and the corresponding absorption coefficients to obtain the final exponent 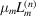 in equation (3). Finally, the calculation of the absorption factor *A*_**h**_ for the reflection **h** involves summing 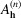 for all crystal voxels, as shown in equation (2).

In a tomography reconstruction, there are air/vacuum regions outside of the crystal sample which contribute negligible absorption effect. It could be argued that it may save computational time if they are neglected. However, as illustrated in Fig. 2 ▸, if the traversal chooses to stop at the vacuum/air region, the absorption caused by the liquor and the loop at the end of the ray cannot be counted. This is why we choose to stop the traversal at the model’s boundary instead of any vacuum/air region, trading computational efficiency to reduce the impact of segmentation artefacts.

### 2.3. Sampling

**Figure 3:**
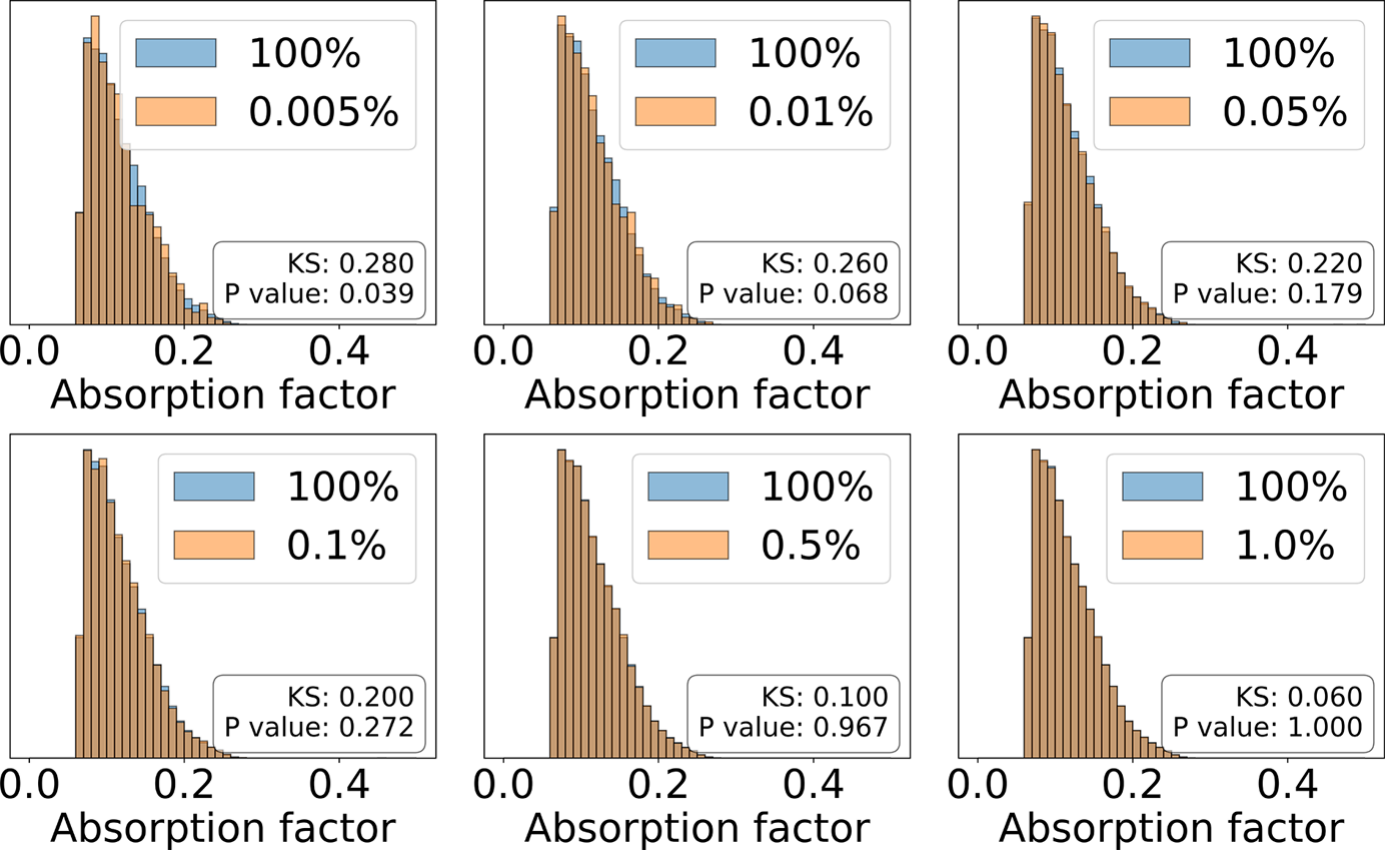
Histograms of absorption factors for different systematic sampling ratios (orange) of a random reflection of thaumatin, compared with that of no-sampling (blue). The overlapping areas of the no-sampling and sampled histograms are shown in dark orange. When the ratio rises to 0.5%, the *P* values of the KS test (Massey, 1951 ▸) become greater than 0.95, failing to reject the null hypothesis, and the two histograms mostly overlap.

To accurately calculate an absorption correction, a precise 3D representation of the sample is essential. This requires a high-resolution tomographically reconstructed volume composed of a large number of voxels. The computing cost associated with calculating path lengths for a large number of crystal voxels is high; along with this, neighbouring diffracting crystal voxels contribute very similar amounts to the overall absorption factor *A*_**h**_. Given this, sub-sampling the crystal voxels can yield potential computational performance increases. On the other hand, equation (2) defines *A*_**h**_ to be the numerical mean of the linear absorption factors of overall crystal voxels. Hence a reduction in the number of summation terms can be obtained by selecting sample crystal voxels whose absorption factor coincides with the average absorption factor of neighbouring crystal voxels. Crystals have a considerable level of structural homogeneity and symmetry, resulting in close absorption effects for adjacent crystal areas. To demonstrate this, in Fig. 3 ▸, six histograms are created to depict the absorption factors for different systematic sampling ratios of a random reflection of thaumatin. The Kolmogorov–Smirnov (KS) test (Massey, 1951 ▸) is employed to evaluate how similar the distribution of each sampling ratio is to the distribution at full sampling (100%). The null hypothesis for this test assumes that the distributions being compared are identical. As the sampling ratio increases, the KS test values decrease and the *P* values increase, suggesting that the sampled distributions become more similar to the full, no-sampling distribution. Notably, *P* values exceed 0.95 starting from a sampling ratio of 0.5%, indicating insufficient evidence to reject the null hypothesis. At a sampling ratio of 1%, the *P* value reaches 1, further affirming this trend.

Hence, employing systematic sampling techniques to select voxels is a viable strategy. This methodology can effectively ensure comprehensive coverage of various crystal locations, facilitating a holistic understanding of the crystal’s absorption characteristics. Conversely, this approach also reduces the influence of specific irregularities or disturbances in a particular area and improves the statistical dependability, yielding more resilient and precise estimations of the absorption factors *A*_**h**_. The methodology employed in our prior research was the utilization of a systematic sampling technique (Lu *et al.*, 2024 ▸). This approach entailed arranging all crystal voxels in a sorted 1D array and selecting a sample for every 2000 crystal voxels (sampling ratio of 0.05%). Nevertheless, it should be noted that the process of systematic sampling may not always result in the selection of the most representative crystal voxels. In order to evaluate the effectiveness of the current systematic sampling, three more sampling approaches are proposed: random sampling, randomized systematic sampling and stratified sampling. The random sampling strategy selects crystal voxels randomly from a uniform distribution (Leal *et al.*, 2008 ▸). In contrast to systematic sampling, randomized systematic sampling selects a crystal voxel randomly within the interval rather than at the edges of the interval. A stratified sampling method is also introduced, which uses a *k*-means clustering approach by starting with a random state. It separates the whole crystal volume into *S* small regions according to their coordinates and the spatial distances to the centroid of the crystal, where *S* is the number of sampled crystal voxels. Then, the sampled crystal voxels are the centroids of the small regions. In Section 3, a comparative evaluation is conducted to evaluate the effectiveness of the sampling strategies in different example datasets.

### 2.4. Ray-tracing by the bisection method

In order to achieve accurate analysis within the 3D model, the standard ray-tracing method must include voxel traversal for every voxel present in the X-ray direction until it meets the end of the tomographic model. In essence, determining the path length requires obtaining information regarding the boundaries of the materials. In macromolecular crystallography experiments, the samples comprise only a small number of components, typically including mother liquor, crystal and loop, and the sizes of these materials are large, exhibiting distant boundaries. Calculating lengths by traversing stepwise along the X-ray path is computationally demanding. In conventional ray-tracing methods, this approach necessitates a large number of repetitive calculations, especially within regions composed of a single material. A more efficient strategy would involve computing distances only across the boundaries separating different materials.

Hence, in order to optimize computational performance, it is beneficial to calculate the boundary coordinates using a bisection approach rather than traversing all of the voxels along both the incident and diffracted X-ray paths. Additionally, a bisection approach has the potential to reduce the time complexity from *O*(*n*) to *O*(log_2_ *n*). The bisection approach is further elaborated in Algorithm 2:
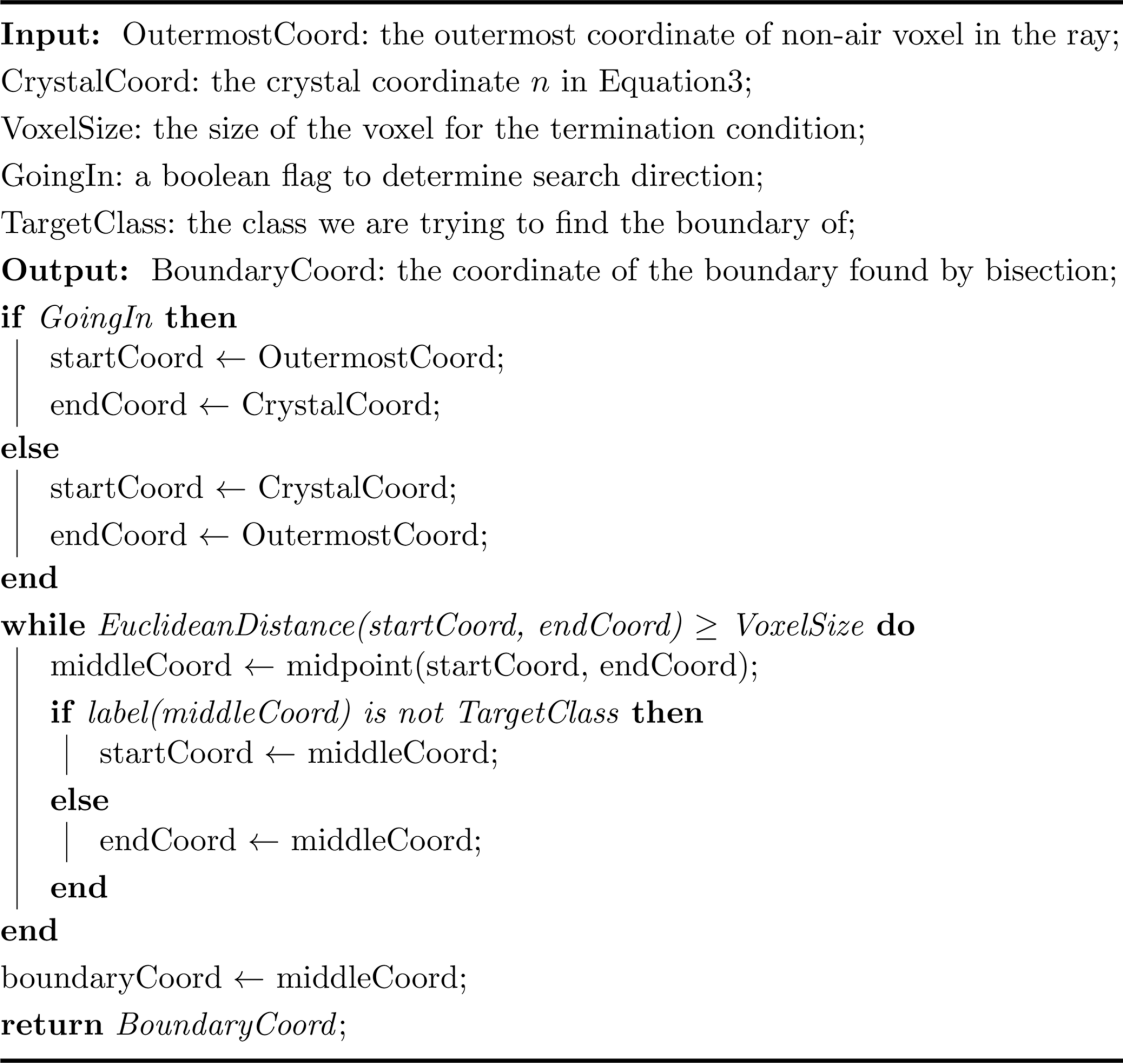


The outermost coordinates are determined by the intersections of the X-rays with the plane of the model, which can be referred to as a cuboid with six planes. These coordinates can be computed using equation (6). The bisection method is capable of determining the coordinates of boundaries; however, it lacks the ability to differentiate between inner and outer limits. Algorithm 2 contains the GoingIn procedure, which enables the bisection method to determine whether the resulting boundaries are classified as inner or outer. Only the crystal’s outer boundary is determined, as the ray traverses from the coordinates within the crystal. This method is based on the assumption that there are no air/vacuum gaps between the crystal and the loop but only liquor in between them. It is sufficient to consider only the borders of the crystal, the loop, and the interface between the sample and the surrounding air or vacuum. The default ordering to determine the boundaries of the bisection method involves the following sequence: the crystal outer boundary, the air boundary (which separates the sample from the air), the loop inner boundary, the loop outer boundary and, subsequently, the boundaries of other materials. The boundary determination process ceases once it has successfully acquired all material boundaries except for the mother liquor. The computation of final path lengths remains consistent with the standard ray-tracing method. The placement of the crystal and the loop can be random, resulting in liquor regions of varying sizes between them that cannot be predetermined. Calculating the dimensions of all these regions is computationally demanding, especially for smaller liquor regions. This variability adds complexity to the computational process. Therefore, the determination of the path length through the mother liquor is the subtraction of the total path length from the path lengths through the crystal, loop, air/vacuum and any additional elements. Hence, if there are some air/vacuum regions inside the sample, the bisection method may incur a high level of inaccuracy.

### 2.5. Gridding interpolation for multiple datasets

**Figure 4:**
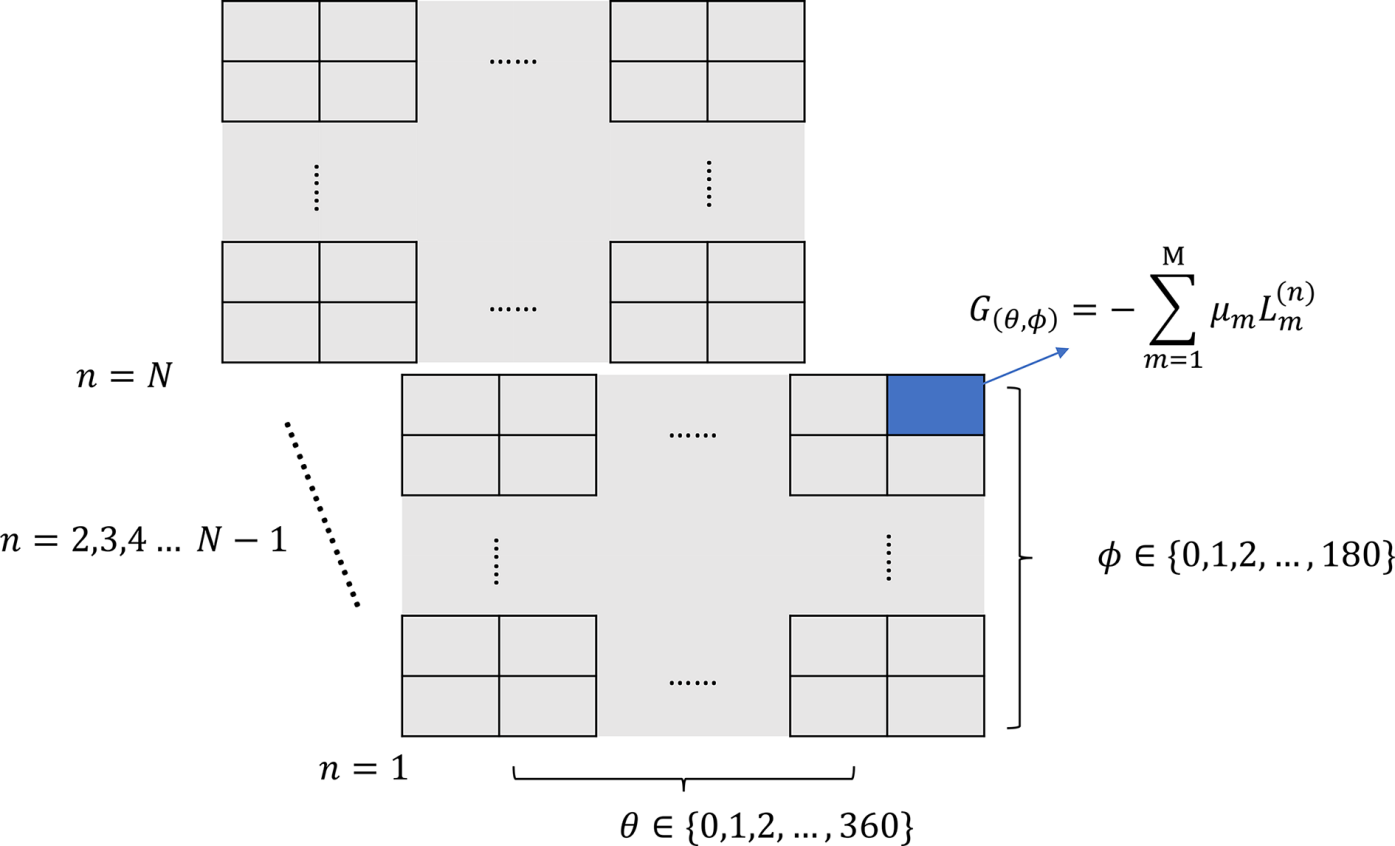
Illustration of gridding interpolation algorithm. There are *N* absorption grids, the same number as the crystal voxels with a shape of (360, 180). Each grid point is an angular-dependent exponent of the absorption factor.

In the aforementioned approaches, the overall computing runtime exhibits linear scaling with the number of reflections, as both the ray-tracing and the bisection method are executed for every individual reflection **h**. When dealing with multiple datasets, the presence of numerous X-rays with similar direction vectors might lead to redundant computations. To enhance computational efficiency, a grid of angular-dependent exponents 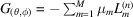 in equation (3) is created by assuming that proximate directional vectors correspond to similar path lengths with the same absorption coefficients. As illustrated in Fig. 4 ▸, this grid is mapped to each crystal coordinate, denoted by *n*. The total number of these path length grids matches the total number of crystal coordinates, indicated as *N*, as defined in equation (2). The path length grids for the crystal coordinates are subsequently computed and retained for future computational purposes. The angular-dependent path length grid has dimensions of (360, 180) with a 1° difference between each grid point. To mitigate the inaccuracy of the edge effect in interpolation, continuity at the edges of the absorption grid is added by concatenating 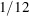 of the data from one side to the other side. Overall, the dimensions of an absorption grid during the interpolation are (420, 210). After constructing the grid, we determine the exponent in the absorption factor 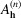 for each crystal coordinate *n* by combining the directional vectors for both the incident and diffracted X-rays. Then, we apply nearest-neighbour interpolation techniques on the grid to complete the calculation.

The absorption factor *A*_**h**_ for the reflection **h** is determined using the same method as described in equation (2). The interpolations are implemented by GSL (a numerical library for C and C++ under the GNU General Public Licence) (Galassi *et al.*, 1996 ▸), which requires double-precision data. If sampling methods are not utilized, the storage of path length grids for all crystal coordinates becomes practically unfeasible due to the significant memory requirements. This is because a double-precision grid for a crystal voxel typically consumes approximately 1 MB of memory. Hence, the utilization of the sampling strategy outlined in the preceding section renders this approach more practicable and feasible.

### 2.6. CUDA implementation

The processing power of GPUs is now widely used in scientific and industrial environments alike, as is perhaps most acutely demonstrated by the breadth and depth of NVIDIA libraries (https://developer.nvidia.com/gpu-accelerated-libraries).

To implement ray-tracing in the *AnACor* package on NVIDIA GPUs, we have used the CUDA programming language. To fully utilize the resources of a GPU, enough parallelism has to be available and exposed by the implementation. For scientific GPUs like NVIDIA A100 GPU or similar, hundreds of thousands of threads need to run concurrently.

There are several ways to expose parallelism in the *AnACor* ray-tracing module. We can parallelize along reflections, incident and diffracted X-rays, and voxels that each ray is stepping through. To achieve a large number of concurrent threads, we have used all of the above. The GPU implementation follows the same steps as outlined in Section 2.2 with modifications as described where necessary.

The major bottleneck of any GPU implementation is the transfer of data from the CPU memory to the GPU memory, as the memory bandwidth between the CPU and GPU is slow. To minimize these transfers, the voxel cube is transferred to the GPU once, and all reflections are calculated in parallel.

After transferring the voxel cube to the GPU, *AnACor* precalculates the rotation of the incident and refracted rays, angles θ and ϕ, and step sizes Δ*xyz* for each reflection. These values are shared by all rays within reflection **h**. Furthermore, to calculate them, a large number of transcendental function calls, which are calculated by the GPU’s special function units that have reduced throughput compared with floating-point units, are used. Then, each ray is calculated by a single threadblock. A threadblock is a set of CUDA threads residing on the same streaming multiprocessor that can share and collaborate on a given task. For each ray, an entry and exit face is determined and threads collaborate on the calculation of an absorption factor 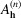 of the ray.

All threads from a threadblock step along the path of the ray in chunks. The number of iterations depends on the size of the voxel cube. Each thread counts the number of different material voxels it encounters. Like the serialized algorithm mentioned previously, it always ends traversal when it encounters the boundary of the overall voxel cube, the reduced operation gathers partial absorption factors calculated by each thread, and the total absorption factor 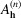 for a given ray is recovered. Also, for better efficiency of memory usage, FP32 is used by each thread.

## 3. Results

**Figure 5:**
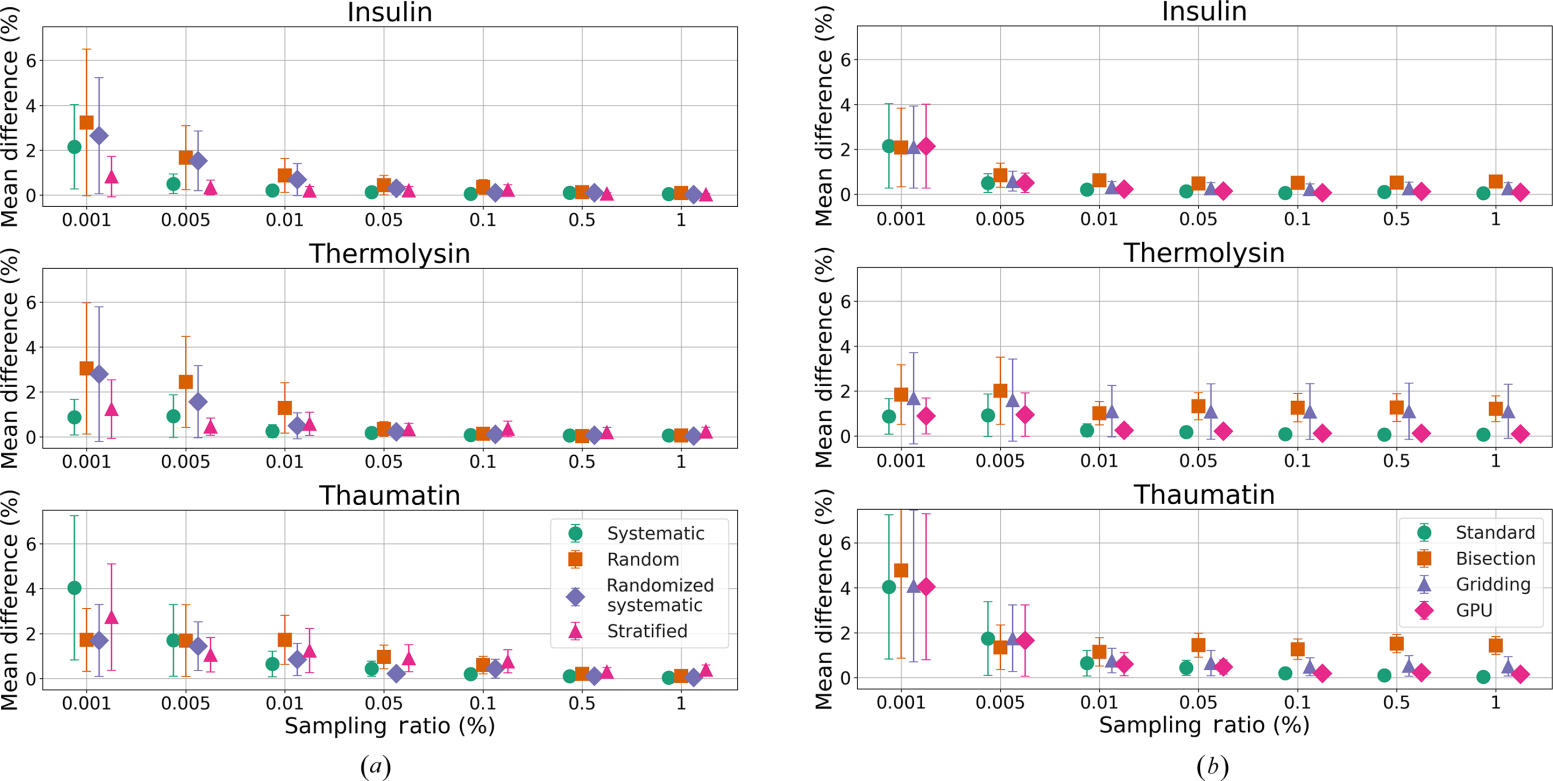
Mean absorption factor differences (%) between sampling and no-sampling (*a*) and between acceleration methods and no-sampling (*b*) for test crystal datasets across various sampling ratios. The sampling methods in (*b*) are all systematic. The error bars represent one standard deviation.

Fig. 5 ▸ compares the mean percentage differences in absorption factors of various sampling strategies (*a*) and acceleration methods (*b*) with no-sampling results. These illustrate the difference across sampling ratios from 0.001% to 1%, with error bars representing one standard deviation.

Fig. 5 ▸(*a*) shows that, when the sampling ratio is at least 0.01%, all mean differences are smaller than 2%, with systematic sampling generally having smaller mean differences and less deviation. After the sampling ratio exceeds 0.5%, the differences become nearly zero across all sampling methods, with minor deviations. The exceptions are stratified sampling in thaumatin and thermolysin. This finding aligns with the KS test results in Fig. 3 ▸, where the *P* value approaches 1 for the 0.5% and 1.0% sampling ratios.

Randomized systematic sampling closely aligns with systematic sampling but shows greater variances. However, it consistently outperforms random sampling for ratios larger than 0.01%. For insulin crystals, which are roughly spherical, stratified sampling proves more effective, showing smaller differences at low sampling ratios. In contrast, for thermolysin and thaumatin crystals, which grow as large rods and bi­pyramids, respectively, stratified sampling performs less favourably at high sampling ratios. This highlights the significant impact of crystal size and shape on the success of stratified sampling strategies.

In this analysis of acceleration techniques for computational sampling, we focus on the systematic sampling method because its absorption factor differences have smaller deviations across various sampling ratios, relying less on the crystal shape. Fig. 5 ▸(*b*) illustrates the absolute mean differences between results from no-sampling and those achieved using acceleration methods. The bisection and gridding methods exhibit consistent differences once the sampling ratio exceeds 0.01%, showing uniform deviations across different datasets. Conversely, the GPU acceleration method, which uses a technique similar to the standard method, displays smaller deviations. Specifically, in the cases of insulin and thaumatin, bisection methods show higher deviations than gridding. However, for thermolysin, gridding results in larger deviations, suggesting that the performance differences between bisection and gridding depend on the crystal’s shape.

**Figure 6:**
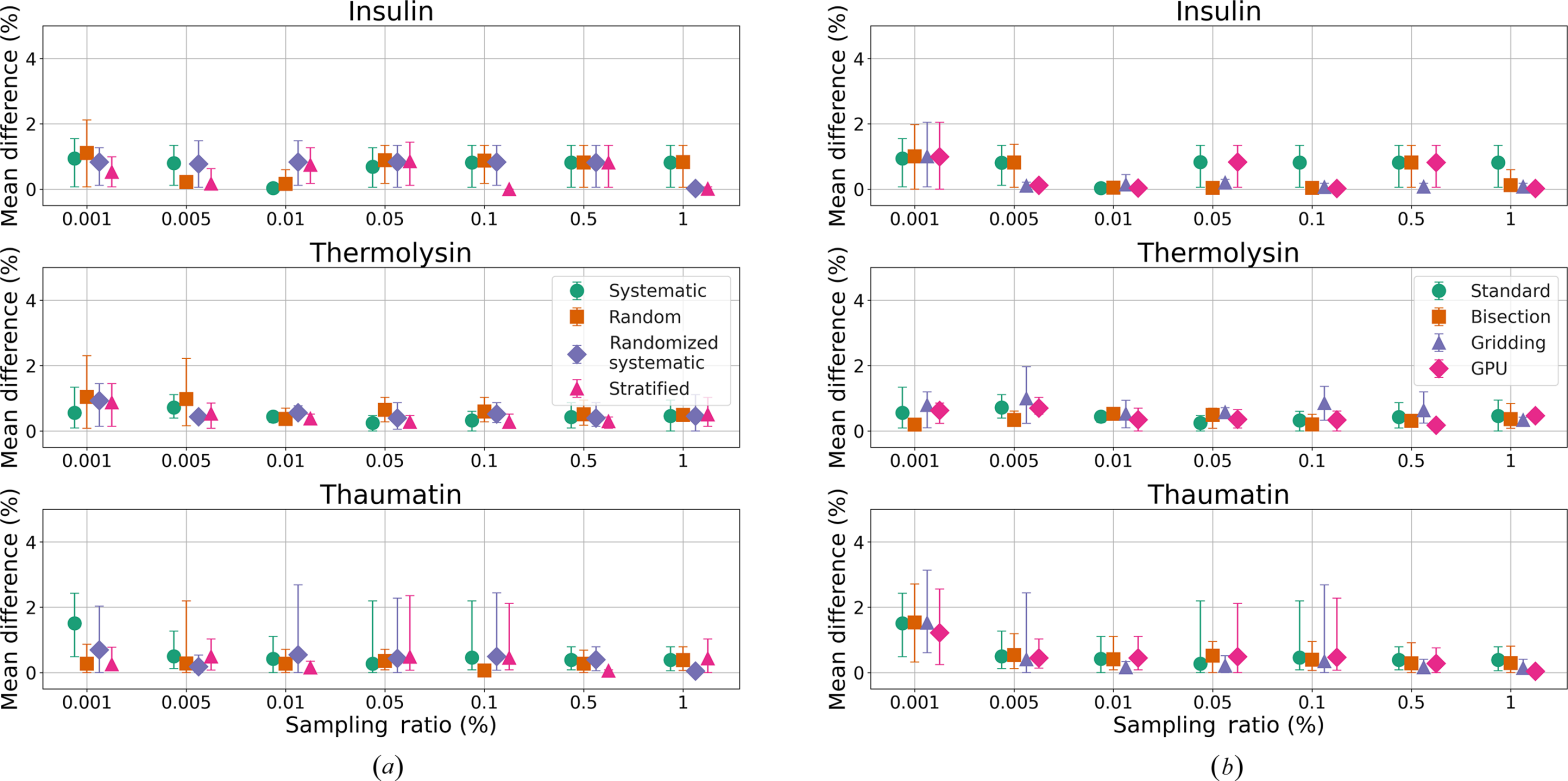
Mean anomalous peak height differences (%) of sulfur atoms between sampling and no-sampling (*a*) and between acceleration methods and no-sampling (*b*) for test crystal datasets across various sampling ratios. The error bars represent the maximum and minimum differences.

Absolute percentage differences between the anomalous peak heights of no-sampling and those determined by sampling and acceleration methods are displayed in Fig. 6 ▸, showing the mean differences of sulfur atoms with peak heights above 10σ. Detailed results for each method are provided in the supporting information. For sampling ratios larger than 0.01%, all sampling and acceleration methods display mean peak height differences within 1% compared with the no-sampling results. This indicates that methods with sampling ratios above 0.01% can achieve similar peak height results. Except for insulin, all sampling methods perform similarly at the same sampling ratios. Although there are differences in the absorption factors of the bisection and gridding methods, their peak height differences perform similarly to those of the standard and GPU methods at the same sampling ratios, except for the gridding method of thermolysin. For insulin, for the thresholds of 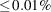, the differences between sampling methods either stabilize around the mean of 1% with similar maximum and minimum values across various sampling ratios or become very close to zero. Conversely, the large differences in absorption factors in the gridding method do not affect the peak heights shown in Fig. 6 ▸(*b*). The anomalous peak height differences remain around 1% with much smaller error bars. Thaumatin shows the largest error bar for sampling ratios of 0.05% and 0.1%, with the maximum absolute differences exceeding 2% for sulfur atom SG_A:CYS66, while the mean differences remain very small.

**Figure 7:**
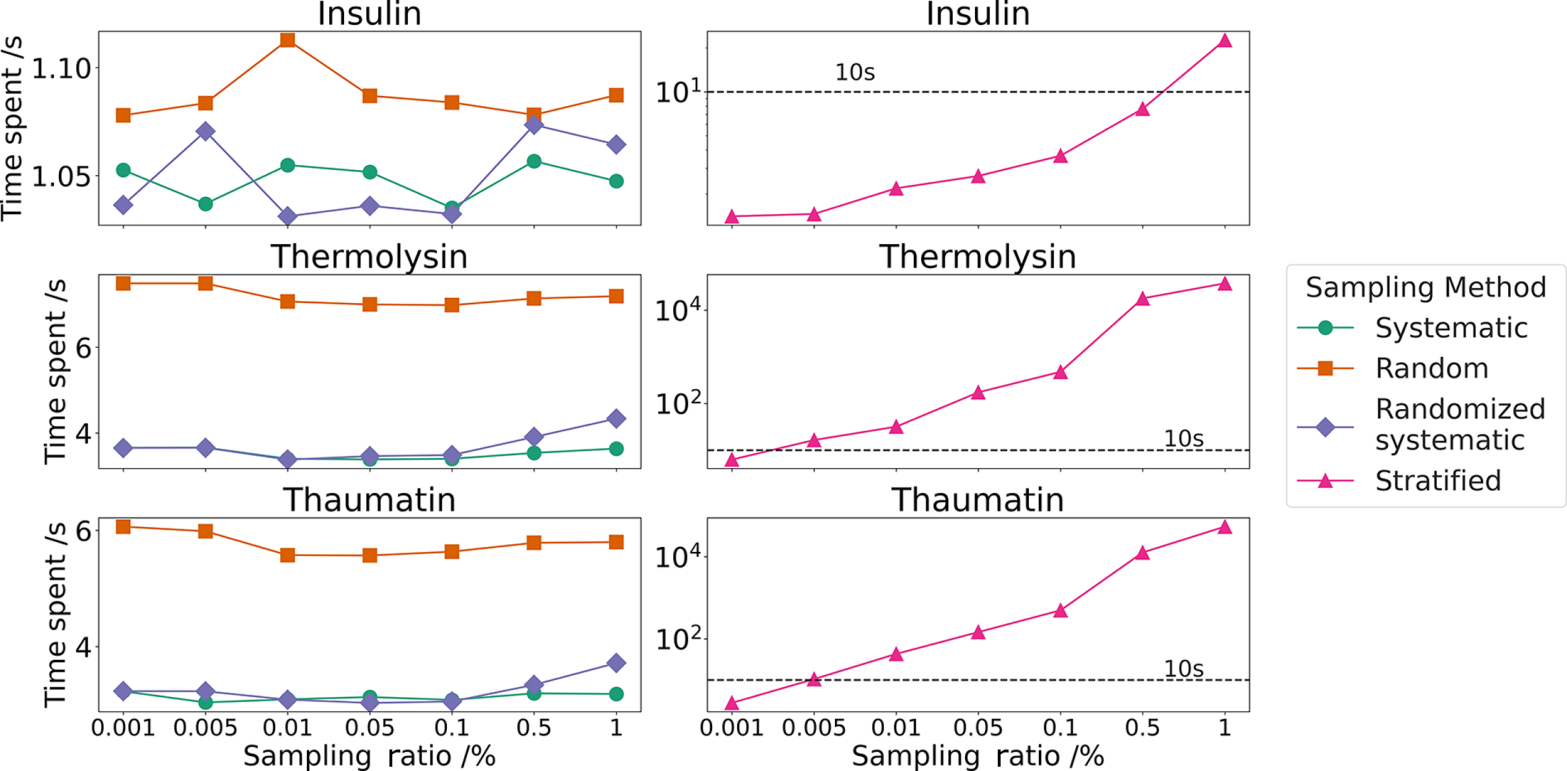
Average time spent on processing sampling methods for 10 runs. They are all determined on the same node with an Intel Xeon Platinum 8268 CPU with 48 cores.

As shown in Fig. 7 ▸, the processing time for systematic and randomized systematic sampling is generally shorter and shows little difference compared with random sampling. Both systematic and randomized systematic sampling times increase with higher sampling ratios, while the random sampling time remains unaffected by the sampling ratios. Although more crystal voxels lead to longer processing times for all sampling methods, they still remain under 10 s. In contrast, the time required for stratified sampling increases exponentially with higher sampling ratios. This is particularly evident for thermolysin and thaumatin crystals, which have substantially more voxels (>30 million and >20 million, respectively) than insulin crystals (>2 million voxels). This variation highlights the impact of crystal shape and sampling strategy on the efficiency of the sampling process.

**Figure 8:**
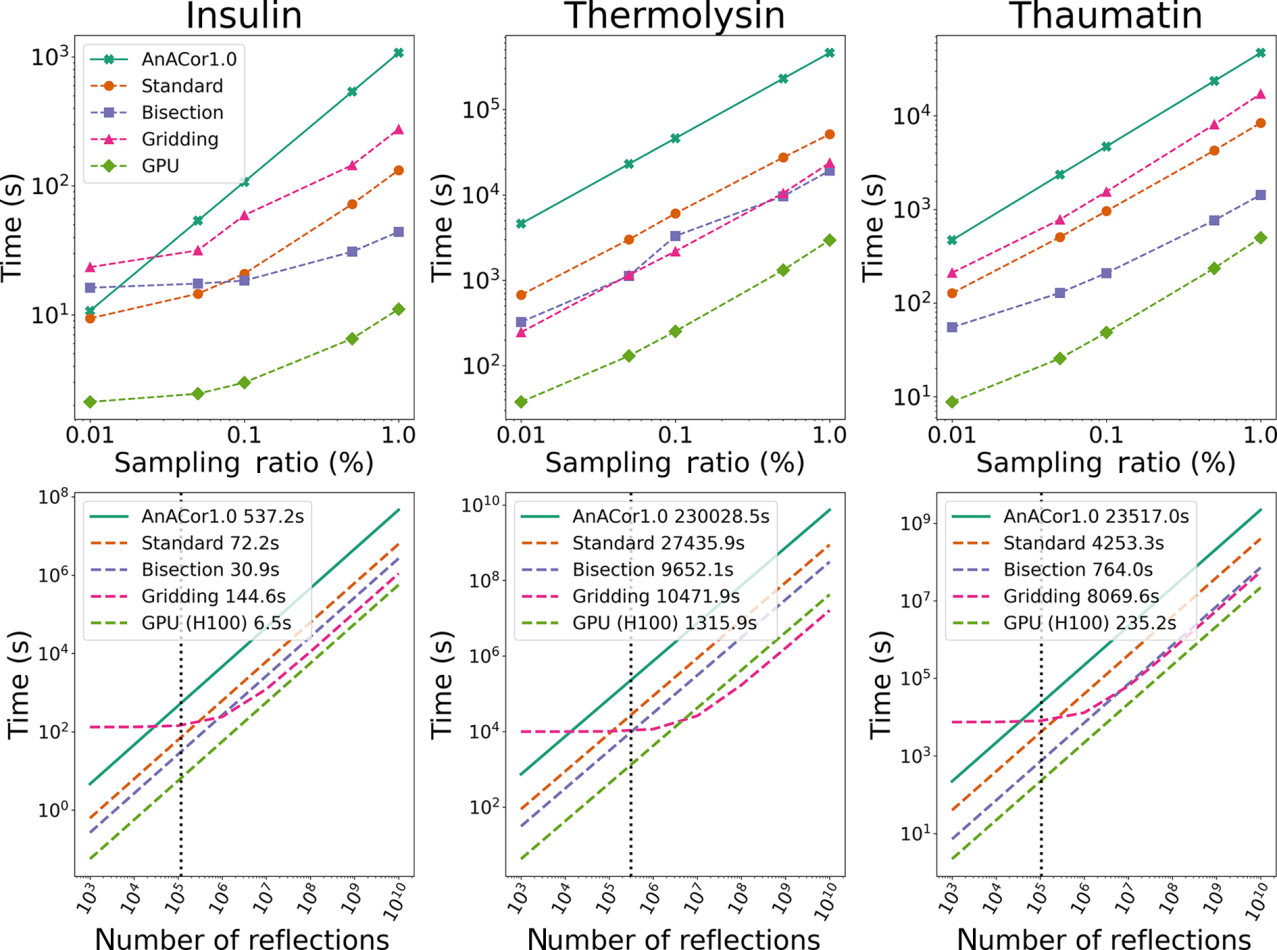
Top: computational time taken by different acceleration methods across sampling ratios. Bottom: computational time taken by acceleration methods with a systematic sampling ratio of 0.5% to process increasing numbers of reflections. The black dotted line indicates the number of reflections in each experimental dataset, with computational times presented in the legend.

**Figure 9:**
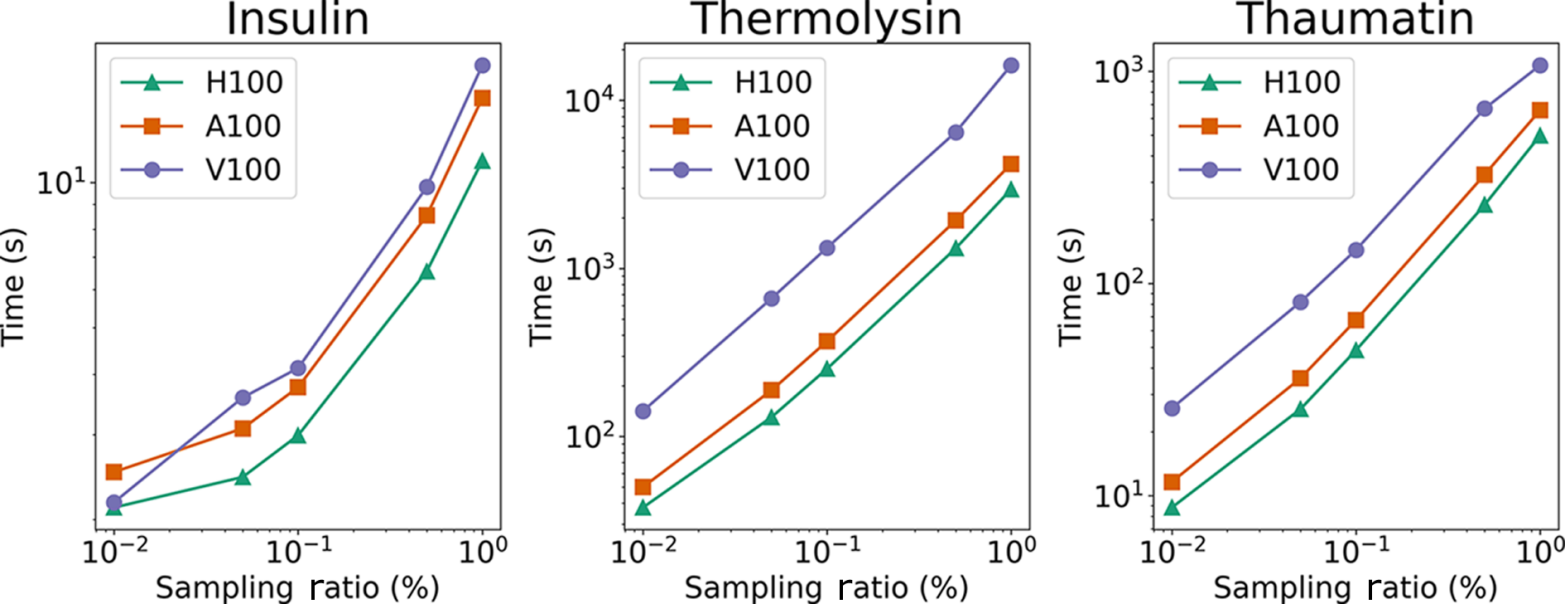
Computational time taken by different NVIDIA computational accelerator cards.

In analysing the balance between accuracy and computational speed provided by acceleration methods, Fig. 8 ▸ presents detailed comparisons of computational time, using a sampling ratio of 0.5%. Fig. 8 ▸ highlights two key findings: firstly, acceleration methods significantly shorten computational times across sampling ratios compared with the baseline reported by Lu *et al.* (2024 ▸); secondly, it evaluates the efficiency in handling an increased number of reflections. The comparative analysis on the top row of Fig. 8 ▸ reveals that acceleration techniques boost computational speed by at least 5 times at sampling ratios >0.01%. Among these, the GPU-based approach stands out for its exceptional time efficiency, taking around 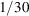, 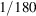 and 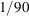 of the time required by the baseline for insulin, thermolysin and thaumatin, respectively. On CPUs, the bisection method demonstrates superior speed over the standard and gridding methods for sampling ratios >0.1%. Notably, the performance of the gridding method varies with sample shape and the number of reflections: it is faster for thermolysin, while for insulin and thaumatin, the gridding method is less efficient than the standard method. The lower section of Fig. 8 ▸ illustrates cross-over points between the gridding method and other CPU-based methods. Notably, with an increasing number of reflections, as seen in thermolysin and thaumatin datasets, the performance of the gridding method approaches that of the GPU method. This emphasizes that the gridding method has the advantage when dealing with large datasets if advanced GPUs are not available. Fig. 9 ▸ compares the performance of three modern NVIDIA computational cards, H100, A100 and V100, across different sampling ratios. The results indicate similar trends in time expenditure among these GPU models, highlighting the consistent benefits of GPU acceleration across a range of computational hardware. This comparison underscores the importance of choosing the right acceleration method and computational hardware based on the specific requirements of the diffraction experiment, balancing speed against the need for accuracy in the final results.

## 4. Discussion

In this study, we demonstrate the effectiveness of acceleration methods in *AnACor2.0* for improving the efficiency of analytical absorption corrections over *AnACor1.0* (Lu *et al.*, 2024 ▸). We detail the utilization of a ray-tracing algorithm for path length calculations in tomography reconstructions. This method is versatile and applicable to various fields for the determination of X-ray diffraction path lengths when a 3D model and its orientation are available.

In the previous study (Lu *et al.*, 2024 ▸), we demonstrated the validity of the ray-tracing approach for analytical absorption corrections based on long-wavelength data from proteins crystallized in monoclinic and triclinic space groups. The data presented here, from crystals in cubic (insulin), hexagonal (thermolysin) and tetragonal (thaumatin) space groups, confirm the previous findings. We show that a combination of analytical absorption correction and spherical harmonics yields substantial improvements in data quality, over spherical harmonics, for data collected at wavelengths larger than 3.5 Å (supporting information).

Our findings indicate that the systematic sampling used in *AnACor1.0* consistently yields stable results with minimal differences and variance compared with no-sampling approaches, across increasing sampling ratios. Interestingly, stratified sampling, employing a *k*-means clustering algorithm, can outperform systematic sampling for crystals with more spherical shapes, such as insulin, even for small sampling ratios. However, it becomes less practical for crystals with a large number of voxels due to the exponentially increasing sampling time and the challenges of achieving global optimization with the clustering algorithm. Random and randomized systematic sampling do not show clear advantages over the other sampling methods, so they are removed from practical use in *AnACor2.0*. While stratified sampling is recommended for small crystals and spherical crystals, systematic sampling remains the default option in *AnACor2.0*.

The analysis also reveals that the deviations between sampled and no-sampling absorption factors diminish beyond a 0.5% sampling ratio threshold in the test crystal datasets, advocating for the use of sampling-based calculations. For all the sampling methods, the anomalous peak height results, for sampling ratios larger than 0.01%, show mean differences smaller than 1% compared with no-sampling. The default sampling ratio is set to 0.5% to ensure accuracy, as confirmed by the *P* value of the KS test of over 0.95. To prioritize computational speed, the sampling ratio can be adjusted accordingly.

The introduction of acceleration techniques in this study has led to a remarkable increase in computational efficiency, improving the performance up to 175 times over *AnACor1.0* (Lu *et al.*, 2024 ▸), by using NVIDIA GPUs. Although the standard and GPU methods use the same underlying algorithm, the GPU method enhances data transfer by employing float32 in core computations. This optimization addresses the significant bottleneck of transferring data from the CPU to the GPU. GPU acceleration with NVIDIA’s H100 and a sampling ratio of 0.5% reduce the processing time of insulin and thaumatin to a few minutes (Fig. 8 ▸), maintaining an absorption factor difference below 0.5%, compared with 100% sampling results (Fig. 5 ▸).

If GPU acceleration is not available, *AnACor2.0* also offers the bisection and gridding methods for improved CPU performance.

The bisection method emerges as a fast option, reducing the time complexity from *O*(*n*) to *O*(log_2_ *n*). Meanwhile, the gridding method is particularly adept at handling large datasets, offering an interpolation approach to reduce computational time. The results reveal that the bisection algorithm consistently shows the largest differences from no-sampling outcomes in the insulin and thaumatin cases. This is attributed to its approximation approach to the standard ray-tracing method, which assumes fixed relative locations for different materials and significant spacing between their boundaries. The gridding method enhances computational efficiency by pre-calculating absorption factors in a spherical coordinate system with 1° increments between grids and employing nearest-neighbour interpolation during the inference stage. The efficiency of the gridding method surpasses that of other methods when the number of reflections reaches a certain threshold, as depicted in Fig. 8 ▸, but it is sample dependent. For instance, in the case of insulin and thaumatin, with a smaller number of reflections, the advantage of the gridding method is reduced, as fewer computations of similar path lengths are needed. However, the gridding method can introduce errors because of the nearest-neighbour interpolation when there is a large path length difference between adjacent gridding points, as illustrated in the thermolysin case. In Fig. 2 ▸, if the direction of the ray rotates anticlockwise, the path lengths through the solvent increase significantly, causing inaccurate interpolation. Therefore, the gridding method is more suitable for cases where the number of reflections is large and the crystal shape is closer to spherical.

The mean absorption factor differences between the bisection and gridding methods are larger than those of GPU acceleration. However, as illustrated in Fig. 6 ▸(*b*), this is not reflected in the anomalous peak height differences of sulfur atoms, which are similar across the acceleration methods. Hence, a user can benefit from any of the acceleration methods presented, with their choice determined by the computational hardware available to them.

## 5. Conclusion

Analytical absorption corrections based on a ray-tracing approach improve the data quality for macromolecular crystallography at very long wavelengths. *AnACor2.0* leverages numerical algorithms and GPU parallelism to significantly increase computational efficiency for calculating analytical absorption factors.

*AnACor2.0* can calculate absorption factors in 6.5 s for insulin, 1315.9 s for thermolysin, and 235.2 s for thaumatin, with GPU acceleration. This achieves computational efficiency improvements of over 90×, 175× and 100×, respectively, compared with *AnACor1.0*. The deviations in absorption factors are minimal compared with the no-sampling results with *AnACor1.0*’s standard ray-tracing method, at 0.09% for insulin and thermolysin, and 0.16% for thaumatin. Additionally, the mean anomalous peak heights of sulfur atoms show deviations of only 0.82% for insulin, 0.17% for thermolysin and 0.28% for thaumatin. In our research, the segmented 3D model was created using X-ray tomography at beamline I23, Diamond Light Source. Importantly, *AnACor2.0*’s utility extends beyond data from this source and will be integrated within *DIALS* in the future. It can facilitate analytical absorption corrections for any dataset, provided that a voxel-annotated file is available and the relationship between the coordinate system of the 3D model and the diffraction experiment is clearly defined.

## Supplementary Material

Supplementary tables. DOI: 10.1107/S1600576724009506/yr5139sup1.pdf

## Figures and Tables

**Table 1:** Absorption coefficients of materials in the samples

Sample	Crystal	Liquor	Loop
Insulin at λ = 3.10 Å	0.00745	0.00720	0.00690
Thermolysin at λ = 3.53 Å	0.01312	0.01583	0.01172
Thaumatin at λ = 4.13 Å	0.01926	0.02019	0.01864
