# Supplementary material for: AnACor2.0: a GPU-accelerated open-source software package for analytical absorption corrections in X-ray crystallography
Source: J Appl Crystallogr. 2024 Nov 4;57(Pt 6):1984–95. doi: 10.1107/S1600576724009506 (PMC11611279; doi:10.1107/S1600576724009506)
Supplement: Supplementary file 1 [file j-57-01984-sup1.pdf]

JOURNAL OF APPLIED CRYSTALLOGRAPHY

Volume 57 (2024)

Supporting information for article:

*AnACor*2.0: a GPU-accelerated open-source software package for analytical absorption corrections in X-ray crystallography

Yishun Lu, Karel Adámek, Tihana Stefanic, Ramona Duman, Armin Wagner and Wesley Armour

S1 Merging statistics

This section includes the detailed merging statistics tables of three test crystals after applying sampling methods (S1, S3, and S5) and acceleration methods (S2, S4, and S6), Insulin, Thermolysin and Thaumatin. Three scaling methods are compared: (no correction (No), spherical harmonics correction (SH) [1], and combination of analytical absorption correction and spherical harmonics correction (ACSH) [2]. The abbreviations in the method column in S1, S3, and S5 are Sy: Systematic sampling; R: Random sampling; RS: Randomised Systematic sampling; St: Stratified sampling. The abbreviations in the method column in S2, S4, and S6 are S: Standard method; Bi: Bisection method; G: Gridding method; GPU: CUDA implementation.

Table S1: Merging statistics of Insulin of different sampling methods

	No	SH	ACSH	ACSH	ACSH	ACSH	ACSH	ACSH	ACSH	ACSH	Method
Ratio (%)			100	1	0.5	0.1	0.05	0.01	0.005	0.001	
Wavelength	3.1Å (4keV)										
Resolution (Å) range	55.26 - 2.05 (2.09 - 2.05)										
Space group	$P2_13$										
Unit cell	(78.1469, 78.1469, 78.1469, 90, 90, 90)										
Multiplicity	22.5 (9.0)	22.4 (9.0)	22.4 (9.0)	22.4 (9.0)	22.4 (9.0)	22.4 (9.0)	22.4 (9.0)	22.4 (9.0)	22.4 (9.0)	22.4 (9.0)	Sy, R, RS, St
Completeness	100.00% (98.35%)	100.00% (98.35%)	100.00% (98.35%)	100.00% (98.35%)	100.00% (98.35%)	100.00% (98.35%)	100.00% (98.35%)	100.00% (98.35%)	100.00% (98.35%)	100.00% (98.35%)	Sy, R, RS, St
Observations	114711 (2139)	114428 (2139)	114453 (2139)	114454 (2139)	114454 (2139)	114454 (2139)	114454 (2139)	114452 (2139)	114453 (2139)	114443 (2139)	Sy
				114454 (2139)	114454 (2139)	114453 (2139)	114453 (2139)	114452 (2139)	114451 (2139)	114462 (2139)	R
				114453 (2139)	114454 (2139)	114454 (2139)	114454 (2139)	114453 (2139)	114452 (2139)	114456 (2139)	RS
				114453 (2139)	114454 (2139)	114453 (2139)	114454 (2139)	114454 (2139)	114452 (2139)	114452 (2139)	St
Unique reflections	5106 (238)	5106 (238)	5106 (238)	5106 (238)	5106 (238)	5106 (238)	5106 (238)	5106 (238)	5106 (238)	5106 (238)	Sy, R, RS, St
Mean I/(I)	13.1 (1.6)	24.2 (1.3)	24.4 (1.3)	24.4 (1.3)	24.4 (1.3)	24.4 (1.3)	24.4 (1.3)	24.4 (1.3)	24.4 (1.3)	24.3 (1.3)	Sy
				24.4 (1.3)	24.4 (1.3)	24.4 (1.3)	24.5 (1.3)	24.5 (1.3)	24.3 (1.3)	23.8 (1.3)	R
				24.4 (1.3)	24.4 (1.3)	24.4 (1.3)	24.4 (1.3)	24.4 (1.3)	24.4 (1.3)	24.3 (1.3)	RS
				24.4 (1.3)	24.4 (1.3)	24.4 (1.3)	24.4 (1.3)	24.4 (1.3)	24.4 (1.3)	24.3 (1.3)	St

Rmerge	0.152 (0.808)	0.093 (0.758)	0.092 (0.760)	0.092 (0.760)	0.092 (0.760)	0.092 (0.760)	0.092 (0.760)	0.092 (0.760)	0.092 (0.760)	0.092 (0.759)	Sy
				0.092 (0.760)	0.092 (0.760)	0.092 (0.760)	0.092 (0.760)	0.092 (0.760)	0.092 (0.759)	0.093 (0.759)	R
				0.092 (0.760)	0.092 (0.760)	0.092 (0.760)	0.092 (0.760)	0.092 (0.759)	0.092 (0.760)	0.092 (0.759)	RS
				0.092 (0.760)	0.092 (0.760)	0.092 (0.760)	0.092 (0.760)	0.092 (0.760)	0.092 (0.760)	0.092 (0.759)	St
Rmeas	0.155 (0.856)	0.095 (0.803)	0.094 (0.805)	0.094 (0.805)	0.094 (0.805)	0.094 (0.805)	0.094 (0.805)	0.094 (0.805)	0.094 (0.805)	0.094 (0.804)	Sy
				0.094 (0.805)	0.094 (0.805)	0.094 (0.805)	0.094 (0.805)	0.094 (0.805)	0.094 (0.804)	0.095 (0.804)	R
				0.094 (0.805)	0.094 (0.805)	0.094 (0.805)	0.094 (0.805)	0.094 (0.805)	0.094 (0.805)	0.094 (0.804)	RS
				0.094 (0.805)	0.094 (0.805)	0.094 (0.805)	0.094 (0.805)	0.094 (0.805)	0.094 (0.805)	0.094 (0.804)	St
Rpim	0.030 (0.281)	0.018 (0.263)	0.018 (0.264)	0.018 (0.264)	0.018 (0.264)	0.018 (0.264)	0.018 (0.264)	0.018 (0.264)	0.018 (0.264)	0.018 (0.264)	Sy
				0.018 (0.264)	0.018 (0.264)	0.018 (0.264)	0.018 (0.264)	0.018 (0.264)	0.018 (0.263)	0.018 (0.263)	R
				0.018 (0.264)	0.018 (0.264)	0.018 (0.264)	0.018 (0.264)	0.018 (0.264)	0.018 (0.264)	0.018 (0.263)	RS
				0.018 (0.264)	0.018 (0.264)	0.018 (0.264)	0.018 (0.264)	0.018 (0.264)	0.018 (0.264)	0.018 (0.263)	St
CC½	0.998 (0.618)	1.000 (0.764)	1.000 (0.763)	1.000 (0.763)	1.000 (0.763)	1.000 (0.763)	1.000 (0.763)	1.000 (0.763)	1.000 (0.763)	0.999 (0.765)	Sy
				1.000 (0.763)	1.000 (0.763)	1.000 (0.763)	1.000 (0.763)	1.000 (0.763)	1.000 (0.764)	0.999 (0.764)	R
				1.000 (0.763)	1.000 (0.763)	1.000 (0.763)	1.000 (0.763)	1.000 (0.764)	1.000 (0.763)	1.000 (0.764)	RS
				1.000 (0.763)	1.000 (0.763)	1.000 (0.763)	1.000 (0.763)	1.000 (0.763)	1.000 (0.763)	1.000 (0.764)	St
Anomalous slope	0.819	0.881	0.864	0.864	0.864	0.865	0.865	0.865	0.863	0.858	Sy
				0.864	0.865	0.866	0.866	0.865	0.862	0.851	R
				0.864	0.864	0.864	0.864	0.864	0.865	0.857	RS
				0.864	0.864	0.864	0.864	0.865	0.864	0.859	St
Reflections used for Rwork	4860	4860	4860	4860	4860	4860	4860	4860	4860	4860	Sy, R, RS, St
Reflections used for Rfree	234	234	234	234	234	234	234	234	234	234	Sy, R, RS, St
Rwork	0.1771	0.1773	0.1772	0.1772	0.1772	0.1772	0.1772	0.1772	0.1772	0.1772	Sy
				0.1772	0.1772	0.1772	0.1772	0.1772	0.1775	0.1776	R
				0.1772	0.1772	0.1772	0.1772	0.1772	0.1772	0.1772	RS
				0.1772	0.1772	0.1772	0.1772	0.1772	0.1771	0.1772	St
Rfree	0.2347	0.2255	0.2258	0.2259	0.2258	0.226	0.2259	0.2252	0.226	0.2254	Sy
				0.226	0.2255	0.2259	0.2259	0.2252	0.2258	0.2256	R
				0.2257	0.2253	0.2254	0.2259	0.2252	0.2258	0.2251	RS
				0.2252	0.2258	0.2258	0.226	0.2253	0.2249	0.2251	St
CCwork	0.9647	0.9687	0.9686	0.9686	0.9686	0.9686	0.9686	0.9686	0.9686	0.9686	Sy
				0.9686	0.9686	0.9686	0.9686	0.9686	0.9685	0.9685	R

				0.9686	0.9686	0.9686	0.9686	0.9686	0.9686	0.9686	RS
				0.9686	0.9686	0.9686	0.9686	0.9686	0.9686	0.9686	St
CCfree	0.9062	0.9273	0.9271	0.9275	0.9274	0.9272	0.9274	0.9273	0.9273	0.9272	Sy
				0.9273	0.9274	0.9274	0.9274	0.9274	0.9269	0.9273	R
				0.9272	0.9276	0.9275	0.9273	0.9274	0.9274	0.9274	RS
				0.9273	0.9274	0.9271	0.9273	0.9274	0.9274	0.9271	St
Rms BondLength	0.0104	0.0123	0.0123	0.0123	0.0123	0.0123	0.0123	0.0123	0.0123	0.0123	Sy
				0.0123	0.0123	0.0123	0.0123	0.0123	0.0123	0.0123	R
				0.0123	0.0123	0.0123	0.0123	0.0123	0.0123	0.0123	RS
				0.0123	0.0123	0.0123	0.0124	0.0123	0.0123	0.0123	St
Rms BondAngle	1.6318	1.7366	1.7367	1.7361	1.7359	1.7368	1.7361	1.7362	1.7366	1.7372	Sy
				1.7365	1.7366	1.7355	1.7356	1.7357	1.7368	1.7317	R
				1.7364	1.7362	1.7362	1.7363	1.7367	1.7355	1.7371	RS
				1.7361	1.7360	1.7362	1.7370	1.7367	1.7365	1.7372	St

Table S2: Merging statistics of Insulin of different acceleration methods

	No	SH	ACSH	ACSH	ACSH	ACSH	ACSH	ACSH	ACSH	ACSH	Method
Ratio (%)			100	1	0.5	0.1	0.05	0.01	0.005	0.001	
Wavelength	3.1Å (4keV)										
Resolution (Å) range	55.26 - 2.05 (2.09 - 2.05)										
Space group	$P2_13$										
Unit cell	(78.1469, 78.1469, 78.1469, 90, 90, 90)										
Multiplicity	22.5 (9.0)	22.4 (9.0)	22.4 (9.0)	22.4 (9.0)	22.4 (9.0)	22.4 (9.0)	22.4 (9.0)	22.4 (9.0)	22.4 (9.0)	22.4 (9.0)	S, Bi, G, GPU
Completeness	100.00% (98.35%)	100.00% (98.35%)	100.00% (98.35%)	100.00% (98.35%)	100.00% (98.35%)	100.00% (98.35%)	100.00% (98.35%)	100.00% (98.35%)	100.00% (98.35%)	100.00% (98.35%)	S, Bi, G, GPU
Observations	114711 (2139)	114428 (2139)	114453 (2139)	114454 (2139)	114454 (2139)	114454 (2139)	114454 (2139)	114452 (2139)	114453 (2139)	114443 (2139)	S
				114453 (2139)	114454 (2139)	114453 (2139)	114453 (2139)	114452 (2139)	114453 (2139)	114460 (2139)	Bi
				114454 (2139)	114454 (2139)	114454 (2139)	114453 (2139)	114453 (2139)	114453 (2139)	114460 (2139)	G
				114453 (2139)	114454 (2139)	114453 (2139)	114454 (2139)	114452 (2139)	114453 (2139)	114460 (2139)	GPU
Unique reflections	5106 (238)	5106 (238)	5106 (238)	5106 (238)	5106 (238)	5106 (238)	5106 (238)	5106 (238)	5106 (238)	5106 (238)	S, Bi, G, GPU
Mean I/(I)	13.1 (1.6)	24.2 (1.3)	24.4 (1.3)	24.4 (1.3)	24.4 (1.3)	24.4 (1.3)	24.4 (1.3)	24.4 (1.3)	24.4 (1.3)	24.3 (1.3)	S
				24.4 (1.3)	24.4 (1.3)	24.4 (1.3)	24.4 (1.3)	24.4 (1.3)	24.4 (1.3)	24.2 (1.3)	Bi
				24.4 (1.3)	24.4 (1.3)	24.4 (1.3)	24.4 (1.3)	24.4 (1.3)	24.4 (1.3)	24.1 (1.3)	G
				24.4 (1.3)	24.4 (1.3)	24.4 (1.3)	24.4 (1.3)	24.4 (1.3)	24.4 (1.3)	24.2 (1.3)	GPU
Rmerge	0.152 (0.808)	0.093 (0.758)	0.092 (0.760)	0.092 (0.760)	0.092 (0.760)	0.092 (0.760)	0.092 (0.760)	0.092 (0.760)	0.092 (0.760)	0.092 (0.759)	S
				0.092 (0.760)	0.092 (0.760)	0.092 (0.760)	0.092 (0.760)	0.092 (0.760)	0.092 (0.760)	0.092 (0.759)	Bi

				0.092 (0.760)	0.092 (0.760)	0.092 (0.760)	0.092 (0.760)	0.092 (0.760)	0.092 (0.760)	0.092 (0.758)	G
				0.092 (0.760)	0.092 (0.760)	0.092 (0.760)	0.092 (0.760)	0.092 (0.760)	0.092 (0.760)	0.092 (0.759)	GPU
Rmeas	0.155 (0.856)	0.095 (0.803)	0.094 (0.805)	0.094 (0.805)	0.094 (0.805)	0.094 (0.805)	0.094 (0.805)	0.094 (0.805)	0.094 (0.805)	0.094 (0.804)	S
				0.094 (0.805)	0.094 (0.805)	0.094 (0.805)	0.094 (0.805)	0.094 (0.805)	0.094 (0.805)	0.094 (0.804)	Bi
				0.094 (0.805)	0.094 (0.805)	0.094 (0.805)	0.094 (0.805)	0.094 (0.805)	0.094 (0.805)	0.094 (0.804)	G
				0.094 (0.805)	0.094 (0.805)	0.094 (0.805)	0.094 (0.805)	0.094 (0.805)	0.094 (0.805)	0.094 (0.804)	GPU
Rpim	0.030 (0.281)	0.018 (0.263)	0.018 (0.264)	0.018 (0.264)	0.018 (0.264)	0.018 (0.264)	0.018 (0.264)	0.018 (0.264)	0.018 (0.264)	0.018 (0.264)	S
				0.018 (0.264)	0.018 (0.264)	0.018 (0.264)	0.018 (0.264)	0.018 (0.264)	0.018 (0.264)	0.018 (0.263)	Bi
				0.018 (0.264)	0.018 (0.264)	0.018 (0.264)	0.018 (0.264)	0.018 (0.264)	0.018 (0.264)	0.018 (0.263)	G
				0.018 (0.264)	0.018 (0.264)	0.018 (0.264)	0.018 (0.264)	0.018 (0.264)	0.018 (0.264)	0.018 (0.263)	GPU
CC½	0.998 (0.618)	1.000 (0.764)	1.000 (0.763)	1.000 (0.763)	1.000 (0.763)	1.000 (0.763)	1.000 (0.763)	1.000 (0.763)	1.000 (0.763)	0.999 (0.765)	S
				1.000 (0.763)	1.000 (0.763)	1.000 (0.763)	1.000 (0.763)	1.000 (0.763)	1.000 (0.763)	1.000 (0.765)	Bi
				1.000 (0.763)	1.000 (0.763)	1.000 (0.763)	1.000 (0.763)	1.000 (0.763)	1.000 (0.763)	1.000 (0.765)	G
				1.000 (0.763)	1.000 (0.763)	1.000 (0.763)	1.000 (0.763)	1.000 (0.763)	1.000 (0.763)	1.000 (0.765)	GPU
Anomalous slope	0.819	0.881	0.864	0.864	0.864	0.865	0.865	0.865	0.863	0.858	S
				0.864	0.865	0.864	0.864	0.862	0.863	0.857	Bi
				0.865	0.865	0.865	0.866	0.866	0.864	0.858	G
				0.864	0.865	0.864	0.865	0.865	0.863	0.857	GPU
Reflections used for Rwork	4860	4860	4860	4860	4860	4860	4860	4860	4860	4860	S, Bi, G, GPU
Reflections used for Rfree	234	234	234	234	234	234	234	234	234	234	S, Bi, G, GPU
Rwork	0.1771	0.1773	0.1772	0.1772	0.1772	0.1772	0.1772	0.1772	0.1772	0.1772	S
				0.1772	0.1772	0.1772	0.1772	0.1772	0.1772	0.1772	Bi
				0.1772	0.1772	0.177	0.1772	0.1772	0.1772	0.1772	G
				0.1772	0.1772	0.1771	0.1772	0.1772	0.1772	0.1772	GPU
Rfree	0.2347	0.2255	0.2258	0.2254	0.2255	0.2254	0.226	0.2256	0.2255	0.2255	S
				0.2257	0.2255	0.2258	0.2258	0.2251	0.2255	0.2249	Bi
				0.225	0.2251	0.2252	0.2256	0.2256	0.2251	0.2256	G
				0.2253	0.2254	0.2258	0.2255	0.2259	0.2252	0.225	GPU
CCwork	0.9647	0.9687	0.9686	0.9686	0.9686	0.9686	0.9686	0.9686	0.9686	0.9685	S
				0.9686	0.9686	0.9686	0.9686	0.9686	0.9686	0.9686	Bi
				0.9686	0.9686	0.9686	0.9686	0.9686	0.9686	0.9686	G
				0.9686	0.9686	0.9686	0.9686	0.9686	0.9686	0.9686	GPU
CCfree	0.9062	0.9273	0.9271	0.9277	0.9275	0.9277	0.9272	0.9274	0.9275	0.9271	S
				0.9272	0.9274	0.9271	0.9271	0.9274	0.9275	0.9276	Bi

				0.9275	0.9274	0.9273	0.9272	0.9272	0.9274	0.9273	G
				0.9273	0.9277	0.9272	0.9274	0.9272	0.9275	0.9275	GPU
Rms BondLength	0.0104	0.0123	0.0123	0.0123	0.0123	0.0123	0.0123	0.0123	0.0123	0.0123	S
				0.0123	0.0123	0.0123	0.0123	0.0123	0.0123	0.0123	Bi
				0.0123	0.0123	0.0123	0.0123	0.0123	0.0123	0.0123	G
				0.0123	0.0123	0.0123	0.0123	0.0123	0.0123	0.0123	GPU
Rms BondAngle	1.6318	1.7366	1.7367	1.7358	1.7364	1.7363	1.7367	1.7355	1.7366	1.7370	S
				1.7363	1.7367	1.7358	1.7364	1.7358	1.7368	1.7363	Bi
				1.7359	1.7366	1.7365	1.7364	1.7360	1.7364	1.7351	G
				1.7353	1.7363	1.7365	1.7368	1.7361	1.7364	1.7361	GPU

Table S3: Merging statistics of Thermolysin of different sampling methods

	No	SH	ACSH	ACSH	ACSH	ACSH	ACSH	ACSH	ACSH	ACSH	Method
Ratio (%)			100	1	0.5	0.1	0.05	0.01	0.005	0.001	
Wavelength	3.53Å (3.5keV)										
Resolution (Å) range	129.26 - 2.31 (2.35 - 2.31)										
Space group	$P6_122$										
Unit cell	(93.0899, 93.0899, 129.259, 90, 90, 120)										
Multiplicity	20.8 (9.4)	21.2 (9.4)	21.3 (9.4)	21.3 (9.4)	21.3 (9.4)	21.3 (9.4)	21.3 (9.4)	21.3 (9.4)	21.3 (9.4)	21.3 (9.4)	Sy, R, RS, St
Completeness	96.47% (90.23%)	96.47% (90.23%)	96.47% (90.23%)	96.47% (90.23%)	96.47% (90.23%)	96.47% (90.23%)	96.47% (90.23%)	96.47% (90.23%)	96.47% (90.23%)	96.47% (90.23%)	Sy, R, RS, St
Observations	301638 (6188)	307756 (6189)	309125 (6189)	309121 (6189)	309121 (6189)	309126 (6189)	309124 (6189)	309128 (6189)	309109 (6189)	309128 (6189)	Sy
				309123 (6189)	309122 (6189)	309123 (6189)	309121 (6189)	309130 (6189)	309138 (6189)	309177 (6189)	R
				309124 (6189)	309121 (6189)	309120 (6189)	309115 (6189)	309125 (6189)	309147 (6189)	309106 (6189)	RS
				309116 (6189)	309116 (6189)	309122 (6189)	309115 (6189)	309111 (6189)	309127 (6189)	309156 (6189)	St
Unique reflections	14513 (656)	14513 (656)	14513 (656)	14513 (656)	14513 (656)	14513 (656)	14513 (656)	14513 (656)	14513 (656)	14513 (656)	Sy, R, RS, St
Mean I/(I)	7.2 (1.1)	11.9 (2.0)	25.5 (5.6)	25.6 (5.7)	25.6 (5.7)	25.5 (5.6)	25.5 (5.6)	25.6 (5.7)	25.1 (5.5)	25.6 (5.7)	Sy
				25.6 (5.7)	25.6 (5.7)	25.6 (5.7)	25.5 (5.6)	25.4 (5.6)	25.0 (5.4)	24.5 (5.2)	R
				25.5 (5.6)	25.6 (5.7)	25.7 (5.7)	25.5 (5.6)	25.6 (5.7)	25.9 (5.8)	25.7 (5.8)	RS
				25.6 (5.7)	25.5 (5.6)	25.5 (5.6)	25.5 (5.6)	25.4 (5.6)	25.5 (5.6)	25.9 (5.8)	St
Rmerge	0.193 (0.405)	0.144 (0.409)	0.102 (0.367)	0.103 (0.366)	0.103 (0.366)	0.102 (0.368)	0.102 (0.366)	0.102 (0.367)	0.102 (0.365)	0.103 (0.367)	Sy
				0.103 (0.367)	0.103 (0.367)	0.103 (0.367)	0.102 (0.367)	0.103 (0.366)	0.102 (0.365)	0.103 (0.367)	R
				0.102 (0.368)	0.102 (0.367)	0.102 (0.367)	0.103 (0.366)	0.102 (0.366)	0.103 (0.368)	0.104 (0.373)	RS
				0.103 (0.366)	0.102 (0.367)	0.102 (0.367)	0.102 (0.367)	0.102 (0.366)	0.102 (0.366)	0.103 (0.369)	St

Rmeas	0.197 (0.427)	0.147 (0.431)	0.105 (0.387)	0.105 (0.386)	0.105 (0.386)	0.105 (0.387)	0.105 (0.386)	0.105 (0.387)	0.105 (0.385)	0.105 (0.387)	Sy
				0.105 (0.386)	0.105 (0.386)	0.105 (0.386)	0.105 (0.387)	0.105 (0.386)	0.105 (0.384)	0.105 (0.386)	R
				0.105 (0.387)	0.105 (0.387)	0.105 (0.386)	0.105 (0.386)	0.105 (0.386)	0.105 (0.388)	0.107 (0.394)	RS
				0.105 (0.386)	0.105 (0.387)	0.105 (0.387)	0.105 (0.386)	0.105 (0.386)	0.105 (0.386)	0.105 (0.389)	St
Rpim	0.040 (0.132)	0.029 (0.133)	0.021 (0.120)	0.021 (0.120)	0.021 (0.120)	0.021 (0.120)	0.021 (0.120)	0.021 (0.120)	0.021 (0.119)	0.021 (0.120)	Sy
				0.021 (0.120)	0.021 (0.120)	0.021 (0.120)	0.021 (0.120)	0.021 (0.120)	0.021 (0.119)	0.021 (0.120)	R
				0.021 (0.120)	0.021 (0.120)	0.021 (0.120)	0.021 (0.120)	0.021 (0.120)	0.021 (0.120)	0.021 (0.122)	RS
				0.021 (0.120)	0.021 (0.120)	0.021 (0.120)	0.021 (0.120)	0.021 (0.120)	0.021 (0.120)	0.021 (0.120)	St
CC½	0.994 (0.925)	0.996 (0.933)	0.998 (0.944)	0.998 (0.944)	0.998 (0.944)	0.998 (0.944)	0.998 (0.944)	0.998 (0.944)	0.998 (0.945)	0.998 (0.944)	Sy
				0.998 (0.944)	0.998 (0.944)	0.998 (0.944)	0.998 (0.944)	0.998 (0.945)	0.998 (0.945)	0.998 (0.945)	R
				0.998 (0.944)	0.998 (0.944)	0.998 (0.944)	0.998 (0.945)	0.998 (0.944)	0.998 (0.944)	0.998 (0.942)	RS
				0.998 (0.944)	0.998 (0.944)	0.998 (0.944)	0.998 (0.944)	0.998 (0.944)	0.998 (0.945)	0.998 (0.943)	St
Anomalous slope	0.208	0.337	0.819	0.825	0.824	0.818	0.818	0.824	0.797	0.83	Sy
				0.823	0.824	0.824	0.821	0.81	0.787	0.762	R
				0.82	0.825	0.829	0.821	0.823	0.837	0.84	RS
				0.826	0.818	0.816	0.82	0.817	0.82	0.844	St
Reflections used for Rwork	13762	13762	13762	13762	13762	13762	13762	13762	13762	13762	Sy, R, RS, St
Reflections used for Rfree	686	686	686	686	686	686	686	686	686	686	Sy, R, RS, St
Rwork	0.1902	0.1833	0.1774	0.1774	0.1774	0.1769	0.1774	0.1776	0.1767	0.1783	Sy
				0.1769	0.1774	0.1774	0.1773	0.1774	0.1772	0.1772	R
				0.1774	0.1774	0.1773	0.1774	0.1774	0.1773	0.177	RS
				0.1773	0.1773	0.1774	0.1772	0.1773	0.1773	0.1772	St
Rfree	0.2219	0.2159	0.211	0.211	0.2108	0.2099	0.211	0.2116	0.2095	0.2139	Sy
				0.2098	0.211	0.2109	0.2106	0.2111	0.2112	0.2114	R
				0.211	0.2109	0.2108	0.2108	0.2109	0.2112	0.2096	RS
				0.2107	0.2108	0.2112	0.211	0.2109	0.211	0.2111	St
CCwork	0.9507	0.9511	0.9526	0.9526	0.9526	0.9527	0.9525	0.9523	0.9528	0.9508	Sy
				0.9527	0.9525	0.9526	0.9526	0.9525	0.9527	0.9527	R
				0.9525	0.9525	0.9526	0.9525	0.9525	0.9526	0.9525	RS
				0.9526	0.9526	0.9526	0.9526	0.9526	0.9525	0.9526	St
CCfree	0.9247	0.9267	0.9289	0.9288	0.9289	0.9295	0.9288	0.9282	0.9299	0.9251	Sy
				0.9295	0.9288	0.9289	0.929	0.9288	0.9289	0.9287	R
				0.9287	0.9289	0.929	0.9289	0.929	0.9286	0.9296	RS
				0.929	0.929	0.9289	0.9287	0.9288	0.9289	0.9288	St
Rms BondLength	0.0071	0.0065	0.0073	0.0073	0.0073	0.0072	0.0073	0.0072	0.0073	0.0072	Sy
				0.0073	0.0073	0.0073	0.0073	0.0072	0.0072	0.0072	R

				0.0072	0.0073	0.0073	0.0073	0.0073	0.0073	0.0073	RS
				0.0073	0.0073	0.0072	0.0073	0.0073	0.0073	0.0072	St
Rms BondAngle	1.6716	1.6481	1.7148	1.7164	1.7151	1.7096	1.7157	1.7105	1.7114	1.7109	Sy
				1.7108	1.7165	1.7159	1.7157	1.7093	1.7123	1.7086	R
				1.7141	1.7150	1.7160	1.7145	1.7135	1.7130	1.7125	RS
				1.7156	1.7141	1.7126	1.7141	1.7146	1.7127	1.7126	St

Table S4: Merging statistics of Thermolysin of different acceleration methods

	No	SH	ACSH	ACSH	ACSH	ACSH	ACSH	ACSH	ACSH	ACSH	Method
Ratio (%)			100	1	0.5	0.1	0.05	0.01	0.005	0.001	
Wavelength	3.53Å (3.5keV)										
Resolution (Å) range	129.26 - 2.31 (2.35 - 2.31)										
Space group	$P6_122$										
Unit cell	(93.0899, 93.0899, 129.259, 90, 90, 120)										
Multiplicity	20.8 (9.4)	21.2 (9.4)	21.3 (9.4)	21.3 (9.4)	21.3 (9.4)	21.3 (9.4)	21.3 (9.4)	21.3 (9.4)	21.3 (9.4)	21.3 (9.4)	S, Bi, G, GPU
Completeness	96.47% (90.23%)	96.47% (90.23%)	96.47% (90.23%)	96.47% (90.23%)	96.47% (90.23%)	96.47% (90.23%)	96.47% (90.23%)	96.47% (90.23%)	96.47% (90.23%)	96.47% (90.23%)	S, Bi, G, GPU
Observations	301638 (6188)	307756 (6189)	309125 (6189)	309121 (6189)	309121 (6189)	309126 (6189)	309124 (6189)	309128 (6189)	309109 (6189)	309128 (6189)	S
				309126 (6189)	309120 (6189)	309122 (6189)	309127 (6189)	309129 (6189)	309105 (6189)	309121 (6189)	Bi
				309095 (6189)	309093 (6189)	309101 (6189)	309103 (6189)	309101 (6189)	309091 (6189)	309109 (6189)	G
				309125 (6189)	309123 (6189)	309127 (6189)	309127 (6189)	309128 (6189)	309109 (6189)	309125 (6189)	GPU
Unique reflections	14513 (656)	14513 (656)	14513 (656)	14513 (656)	14513 (656)	14513 (656)	14513 (656)	14513 (656)	14513 (656)	14513 (656)	S, Bi, G, GPU
Mean I/(I)	7.2 (1.1)	11.9 (2.0)	25.5 (5.6)	25.6 (5.7)	25.6 (5.7)	25.5 (5.6)	25.5 (5.6)	25.6 (5.7)	25.1 (5.5)	25.6 (5.7)	S
				25.4 (5.6)	25.4 (5.6)	25.4 (5.6)	25.4 (5.6)	25.6 (5.7)	25.4 (5.6)	25.6 (5.8)	Bi
				25.3 (5.5)	25.3 (5.5)	25.5 (5.6)	25.4 (5.6)	25.7 (5.7)	25.3 (5.5)	25.5 (5.7)	G
				25.6 (5.7)	25.5 (5.6)	25.5 (5.6)	25.4 (5.6)	25.6 (5.7)	25.2 (5.5)	25.6 (5.7)	GPU
Rmerge	0.193 (0.405)	0.144 (0.409)	0.102 (0.367)	0.103 (0.366)	0.103 (0.366)	0.102 (0.368)	0.102 (0.366)	0.102 (0.367)	0.102 (0.365)	0.103 (0.367)	S
				0.103 (0.367)	0.103 (0.366)	0.103 (0.367)	0.103 (0.366)	0.102 (0.367)	0.102 (0.365)	0.103 (0.366)	Bi
				0.102 (0.367)	0.102 (0.367)	0.103 (0.367)	0.102 (0.367)	0.102 (0.367)	0.102 (0.366)	0.103 (0.368)	G
				0.103 (0.367)	0.102 (0.367)	0.103 (0.367)	0.102 (0.367)	0.102 (0.367)	0.102 (0.365)	0.103 (0.367)	GPU
Rmeas	0.197 (0.427)	0.147 (0.431)	0.105 (0.387)	0.105 (0.386)	0.105 (0.386)	0.105 (0.387)	0.105 (0.386)	0.105 (0.387)	0.105 (0.385)	0.105 (0.387)	S
				0.105 (0.386)	0.105 (0.386)	0.105 (0.386)	0.105 (0.386)	0.105 (0.386)	0.105 (0.384)	0.105 (0.386)	Bi

				0.105 (0.387)	0.105 (0.387)	0.105 (0.386)	0.105 (0.387)	0.105 (0.387)	0.105 (0.386)	0.105 (0.388)	G
				0.105 (0.387)	0.105 (0.387)	0.105 (0.386)	0.105 (0.387)	0.105 (0.386)	0.104 (0.384)	0.105 (0.386)	GPU
Rpim	0.040 (0.132)	0.029 (0.133)	0.021 (0.120)	0.021 (0.120)	0.021 (0.120)	0.021 (0.120)	0.021 (0.120)	0.021 (0.120)	0.021 (0.119)	0.021 (0.120)	S
				0.021 (0.120)	0.021 (0.120)	0.021 (0.120)	0.021 (0.120)	0.021 (0.120)	0.021 (0.119)	0.021 (0.120)	Bi
				0.021 (0.120)	0.021 (0.120)	0.021 (0.120)	0.021 (0.120)	0.021 (0.120)	0.021 (0.119)	0.021 (0.120)	G
				0.021 (0.120)	0.021 (0.120)	0.021 (0.120)	0.021 (0.120)	0.021 (0.120)	0.021 (0.119)	0.021 (0.120)	GPU
CC½	0.994 (0.925)	0.996 (0.933)	0.998 (0.944)	0.998 (0.944)	0.998 (0.944)	0.998 (0.944)	0.998 (0.944)	0.998 (0.944)	0.998 (0.945)	0.998 (0.944)	S
				0.998 (0.944)	0.998 (0.944)	0.998 (0.944)	0.998 (0.944)	0.998 (0.944)	0.998 (0.945)	0.998 (0.944)	Bi
				0.998 (0.945)	0.998 (0.945)	0.998 (0.945)	0.998 (0.944)	0.998 (0.944)	0.998 (0.945)	0.998 (0.944)	G
				0.998 (0.944)	0.998 (0.944)	0.998 (0.944)	0.998 (0.944)	0.998 (0.944)	0.998 (0.945)	0.998 (0.944)	GPU
Anomalous slope	0.208	0.337	0.819	0.825	0.824	0.818	0.818	0.824	0.797	0.83	S
				0.813	0.815	0.816	0.81	0.827	0.819	0.834	Bi
				0.804	0.804	0.82	0.812	0.825	0.807	0.821	G
				0.822	0.818	0.822	0.813	0.823	0.798	0.832	GPU
Reflections used for Rwork	13762	13762	13762	13762	13762	13762	13762	13762	13762	13762	S, Bi, G, GPU
Reflections used for Rfree	686	686	686	686	686	686	686	686	686	686	S, Bi, G, GPU
Rwork	0.1902	0.1833	0.1774	0.1774	0.1774	0.1769	0.1774	0.1776	0.1767	0.1783	S
				0.1773	0.1774	0.1777	0.1774	0.1773	0.1772	0.1788	Bi
				0.1775	0.1774	0.1775	0.1776	0.1776	0.1774	0.1776	G
				0.1774	0.1769	0.1776	0.1773	0.1773	0.1772	0.1786	GPU
Rfree	0.2219	0.2159	0.211	0.211	0.2108	0.2099	0.211	0.2116	0.2095	0.2139	S
				0.2107	0.2108	0.2117	0.2111	0.211	0.211	0.2166	Bi
				0.2112	0.2116	0.2114	0.2114	0.2112	0.2114	0.2108	G
				0.2109	0.2097	0.2118	0.2112	0.211	0.211	0.2155	GPU
CCwork	0.9507	0.9511	0.9526	0.9526	0.9526	0.9527	0.9525	0.9523	0.9528	0.9508	S
				0.9526	0.9526	0.9523	0.9526	0.9526	0.9526	0.9519	Bi
				0.9525	0.9525	0.9524	0.9525	0.9524	0.9525	0.9524	G
				0.9526	0.9527	0.9523	0.9526	0.9526	0.9527	0.9504	GPU
CCfree	0.9247	0.9267	0.9289	0.9288	0.9289	0.9295	0.9288	0.9282	0.9299	0.9251	S
				0.9289	0.9289	0.9282	0.9288	0.9289	0.9289	0.9217	Bi
				0.9287	0.9286	0.9285	0.9285	0.9286	0.9286	0.9289	G
				0.9289	0.9296	0.9282	0.9288	0.9289	0.929	0.9228	GPU
Rms BondLength	0.0071	0.0065	0.0073	0.0073	0.0073	0.0072	0.0073	0.0072	0.0073	0.0072	S
				0.0073	0.0073	0.0072	0.0072	0.0073	0.0073	0.0073	Bi
				0.0073	0.0072	0.0072	0.0072	0.0072	0.0072	0.0073	G
				0.0073	0.0073	0.0072	0.0072	0.0073	0.0072	0.0072	GPU
Rms BondAngle	1.6716	1.6481	1.7148	1.7164	1.7151	1.7096	1.7157	1.7105	1.7114	1.7109	S

				1.7162	1.7173	1.7100	1.7115	1.7135	1.7143	1.7143	Bi
				1.7148	1.7118	1.7137	1.7140	1.7127	1.7112	1.7159	G
				1.7164	1.7140	1.7103	1.7117	1.7128	1.7128	1.7108	GPU

Table S5: Merging statistics of Thaumatin of different sampling methods

	No	SH	ACSH	ACSH	ACSH	ACSH	ACSH	ACSH	ACSH	ACSH	Method
Ratio (%)			100	1	0.5	0.1	0.05	0.01	0.005	0.001	
Wavelength	4.13Å (3keV)										
Resolution (Å) range	150.73 - 2.70 (2.75 - 2.70)										
Space group	$P4_12_12$										
Unit cell	(58.0323, 58.0323, 150.728, 90, 90, 90)										
Multiplicity	13.7<br>(5.4)	13.8<br>(5.4)	13.9<br>(5.4)	13.9<br>(5.4)	13.9<br>(5.4)	13.9<br>(5.4)	13.9<br>(5.4)	13.9<br>(5.4)	13.9<br>(5.4)	13.9<br>(5.4)	Sy, R,<br>RS, St
Completeness	99.21%<br>(90.79%)	99.21%<br>(90.79%)	99.21%<br>(90.79%)	99.21%<br>(90.79%)	99.21%<br>(90.79%)	99.21%<br>(90.79%)	99.21%<br>(90.79%)	99.21%<br>(90.79%)	99.21%<br>(90.79%)	99.21%<br>(90.79%)	Sy
Observations	103768<br>(1873)	104628<br>(1873)	105278<br>(1873)	105277<br>(1873)	105280<br>(1873)	105327<br>(1873)	105277<br>(1873)	105275<br>(1873)	105278<br>(1873)	105341<br>(1873)	Sy, R,<br>RS, St
				105279<br>(1873)	105329<br>(1873)	105275<br>(1873)	105280<br>(1873)	105279<br>(1873)	105272<br>(1873)	105280<br>(1873)	R
				105277<br>(1873)	105280<br>(1873)	105328<br>(1873)	105329<br>(1873)	105276<br>(1873)	105282<br>(1873)	105267<br>(1873)	RS
				105282<br>(1873)	105278<br>(1873)	105328<br>(1873)	105332<br>(1873)	105276<br>(1873)	105275<br>(1873)	105267<br>(1873)	St
Unique reflections	7580<br>(345)	7580<br>(345)	7580<br>(345)	7580<br>(345)	7580<br>(345)	7580<br>(345)	7580<br>(345)	7580<br>(345)	7580<br>(345)	7580<br>(345)	Sy, R,<br>RS, St
Mean I/(I)	19.4<br>(9.0)	24.9<br>(11.1)	58.0<br>(28.9)	58.0<br>(28.9)	57.9<br>(28.8)	57.7<br>(28.9)	57.8<br>(28.7)	58.1<br>(28.9)	58.2<br>(28.9)	52.1<br>(25.6)	Sy
				58.0<br>(28.8)	57.5<br>(28.5)	57.9<br>(28.8)	57.8<br>(28.8)	57.4<br>(28.4)	58.5<br>(29.2)	58.2<br>(28.9)	R
				58.0<br>(28.8)	58.1<br>(29.0)	57.5<br>(28.4)	57.9<br>(29.1)	58.2<br>(29.1)	57.3<br>(28.6)	58.7<br>(29.2)	RS
				57.7<br>(28.6)	57.9<br>(28.9)	57.1<br>(28.2)	57.0<br>(28.1)	57.9<br>(28.9)	58.3<br>(29.1)	59.0<br>(29.5)	St
Rmerge	0.123<br>(0.100)	0.096<br>(0.096)	0.061<br>(0.082)	0.060<br>(0.082)	0.061<br>(0.082)	0.061<br>(0.083)	0.061<br>(0.082)	0.060<br>(0.081)	0.060<br>(0.082)	0.065<br>(0.095)	Sy
				0.060<br>(0.082)	0.061<br>(0.083)	0.061<br>(0.082)	0.061<br>(0.083)	0.061<br>(0.083)	0.060<br>(0.080)	0.060<br>(0.079)	R
				0.060<br>(0.081)	0.060<br>(0.082)	0.061<br>(0.083)	0.061<br>(0.082)	0.060<br>(0.081)	0.061<br>(0.081)	0.060<br>(0.081)	RS
				0.061<br>(0.082)	0.061<br>(0.082)	0.061<br>(0.083)	0.061<br>(0.083)	0.060<br>(0.081)	0.060<br>(0.081)	0.060<br>(0.081)	St
Rmeas	0.128<br>(0.110)	0.099<br>(0.105)	0.063<br>(0.090)	0.063<br>(0.090)	0.063<br>(0.090)	0.063<br>(0.092)	0.063<br>(0.090)	0.063<br>(0.089)	0.063<br>(0.090)	0.068<br>(0.105)	Sy
				0.063<br>(0.090)	0.063<br>(0.092)	0.063<br>(0.090)	0.063<br>(0.091)	0.063<br>(0.091)	0.062<br>(0.088)	0.062<br>(0.087)	R
				0.063<br>(0.089)	0.063<br>(0.090)	0.063<br>(0.091)	0.063<br>(0.091)	0.063<br>(0.089)	0.063<br>(0.089)	0.062<br>(0.088)	RS
				0.063<br>(0.090)	0.063<br>(0.090)	0.063<br>(0.092)	0.063<br>(0.092)	0.063<br>(0.089)	0.063<br>(0.089)	0.062<br>(0.089)	St

Rpim	0.032 (0.044)	0.025 (0.042)	0.016 (0.036)	0.016 (0.036)	0.016 (0.036)	0.016 (0.037)	0.016 (0.036)	0.016 (0.036)	0.016 (0.036)	0.017 (0.042)	Sy
				0.016 (0.036)	0.016 (0.037)	0.016 (0.036)	0.016 (0.037)	0.016 (0.037)	0.016 (0.035)	0.016 (0.035)	R
				0.016 (0.036)	0.016 (0.036)	0.016 (0.037)	0.016 (0.037)	0.016 (0.036)	0.016 (0.036)	0.016 (0.035)	RS
				0.016 (0.036)	0.016 (0.036)	0.016 (0.037)	0.016 (0.037)	0.016 (0.036)	0.016 (0.036)	0.016 (0.036)	St
CC½	0.994 (0.991)	0.995 (0.990)	0.998 (0.993)	0.998 (0.993)	0.998 (0.993)	0.998 (0.992)	0.998 (0.993)	0.998 (0.993)	0.998 (0.993)	0.997 (0.988)	Sy
				0.998 (0.993)	0.998 (0.992)	0.998 (0.993)	0.998 (0.993)	0.998 (0.992)	0.998 (0.993)	0.998 (0.993)	R
				0.998 (0.993)	0.998 (0.993)	0.998 (0.992)	0.998 (0.992)	0.998 (0.993)	0.998 (0.993)	0.998 (0.993)	RS
				0.998 (0.993)	0.998 (0.993)	0.998 (0.992)	0.998 (0.992)	0.998 (0.993)	0.998 (0.993)	0.998 (0.993)	St
Anomalous slope	1.381	1.72	4.142	4.135	4.124	4.137	4.117	4.14	4.145	3.746	Sy
				4.133	4.098	4.129	4.131	4.08	4.172	4.142	R
				4.13	4.147	4.087	4.153	4.157	4.085	4.179	RS
				4.105	4.133	4.056	4.053	4.134	4.159	4.208	St
Reflections used for Rwork	7181	7181	7181	7181	7181	7181	7181	7181	7181	7181	Sy, R, RS, St
Reflections used for Rfree	329	329	329	329	329	329	329	329	329	329	Sy, R, RS, St
Rwork	0.1832	0.1794	0.1721	0.1721	0.1721	0.172	0.1721	0.1718	0.1722	0.1719	Sy
				0.1721	0.1719	0.172	0.1721	0.1722	0.1722	0.1722	R
				0.172	0.1722	0.1718	0.172	0.1721	0.1722	0.1718	RS
				0.1721	0.1721	0.172	0.172	0.1721	0.1721	0.172	St
Rfree	0.2226	0.2207	0.2183	0.2184	0.2181	0.2182	0.2183	0.2181	0.2183	0.2178	Sy
				0.2178	0.2184	0.2183	0.218	0.2182	0.218	0.2187	R
				0.2182	0.2181	0.2182	0.2182	0.2177	0.2181	0.2177	RS
				0.2183	0.2184	0.2181	0.2184	0.2183	0.218	0.2184	St
CCwork	0.9335	0.9343	0.942	0.9422	0.9422	0.9422	0.942	0.9422	0.942	0.9426	Sy
				0.9422	0.9422	0.9421	0.9422	0.942	0.9419	0.9419	R
				0.942	0.9421	0.9423	0.9422	0.9422	0.9421	0.9425	RS
				0.9421	0.9421	0.9422	0.9422	0.9421	0.9421	0.942	St
CCfree	0.8817	0.8808	0.8888	0.889	0.8892	0.8892	0.8887	0.8898	0.8887	0.8905	Sy
				0.8893	0.8892	0.8889	0.8894	0.8892	0.8887	0.8885	R
				0.8888	0.8892	0.8893	0.8892	0.8895	0.8889	0.8897	RS
				0.889	0.8888	0.8894	0.8892	0.8888	0.8893	0.8886	St
Rms BondLength	0.0098	0.0099	0.0095	0.0095	0.0095	0.0095	0.0095	0.0096	0.0095	0.0096	Sy
				0.0095	0.0095	0.0095	0.0095	0.0095	0.0095	0.0095	R
				0.0095	0.0095	0.0095	0.0095	0.0095	0.0095	0.0095	RS
				0.0095	0.0095	0.0095	0.0095	0.0095	0.0095	0.0095	St
Rms BondAngle	1.7533	1.7547	1.7495	1.7522	1.7516	1.7471	1.7495	1.7625	1.7518	1.7501	Sy
				1.7511	1.7473	1.7497	1.7528	1.7519	1.7480	1.7515	R
				1.7501	1.7518	1.7479	1.7471	1.7530	1.7476	1.7524	RS
				1.7519	1.7499	1.7462	1.7475	1.7507	1.7523	1.7529	St

Table S6: Merging statistics of Thaumatin of different acceleration methods

	No	SH	ACSH	ACSH	ACSH	ACSH	ACSH	ACSH	ACSH	ACSH	Method
Ratio (%)			100	1	0.5	0.1	0.05	0.01	0.005	0.001	
Wavelength	4.13Å (3keV)										
Resolution (Å) range	150.73 - 2.70 (2.75 - 2.70)										
Space group	$P4_12_12$										
Unit cell	(58.0323, 58.0323, 150.728, 90, 90, 90)										
Multiplicity	13.7 (5.4)	13.8 (5.4)	13.9 (5.4)	13.9 (5.4)	13.9 (5.4)	13.9 (5.4)	13.9 (5.4)	13.9 (5.4)	13.9 (5.4)	13.9 (5.4)	S, Bi, G, GPU
Completeness	99.21% (90.79%)	99.21% (90.79%)	99.21% (90.79%)	99.21% (90.79%)	99.21% (90.79%)	99.21% (90.79%)	99.21% (90.79%)	99.21% (90.79%)	99.21% (90.79%)	99.21% (90.79%)	S, Bi, G, GPU
Observations	103768 (1873)	104628 (1873)	105278 (1873)	105277 (1873)	105280 (1873)	105327 (1873)	105277 (1873)	105275 (1873)	105278 (1873)	105341 (1873)	S
				105328 (1873)	105331 (1873)	105279 (1873)	105282 (1873)	105277 (1873)	105278 (1873)	105343 (1873)	Bi
				105269 (1873)	105269 (1873)	105267 (1873)	105268 (1873)	105267 (1873)	105266 (1873)	105329 (1873)	G
				105277 (1873)	105326 (1873)	105327 (1873)	105330 (1873)	105276 (1873)	105277 (1873)	105315 (1873)	GPU
Unique reflections	7580 (345)	7580 (345)	7580 (345)	7580 (345)	7580 (345)	7580 (345)	7580 (345)	7580 (345)	7580 (345)	7580 (345)	S, Bi, G, GPU
Mean I/(I)	19.4 (9.0)	24.9 (11.1)	58.0 (28.9)	58.0 (28.9)	57.9 (28.8)	57.7 (28.9)	57.8 (28.7)	58.1 (28.9)	58.2 (28.9)	52.1 (25.6)	S
				57.2 (28.3)	57.4 (28.7)	57.8 (28.8)	57.6 (28.6)	58.0 (28.9)	57.9 (28.7)	51.9 (25.3)	Bi
				58.9 (29.6)	58.7 (29.4)	58.9 (29.6)	58.6 (29.4)	58.9 (29.5)	59.1 (29.6)	52.8 (25.9)	G
				58.0 (28.9)	57.6 (28.9)	57.7 (28.9)	57.1 (28.2)	58.2 (29.0)	58.1 (28.9)	52.3 (25.3)	GPU
Rmerge	0.123 (0.100)	0.096 (0.096)	0.061 (0.082)	0.060 (0.082)	0.061 (0.082)	0.061 (0.083)	0.061 (0.082)	0.060 (0.081)	0.060 (0.082)	0.065 (0.095)	S
				0.061 (0.084)	0.061 (0.084)	0.061 (0.082)	0.061 (0.082)	0.061 (0.082)	0.061 (0.083)	0.065 (0.096)	Bi
				0.060 (0.080)	0.060 (0.080)	0.060 (0.080)	0.060 (0.080)	0.060 (0.080)	0.060 (0.081)	0.064 (0.092)	G
				0.061 (0.082)	0.061 (0.083)	0.061 (0.083)	0.061 (0.084)	0.060 (0.081)	0.060 (0.082)	0.065 (0.090)	GPU
Rmeas	0.128 (0.110)	0.099 (0.105)	0.063 (0.090)	0.063 (0.090)	0.063 (0.090)	0.063 (0.092)	0.063 (0.090)	0.063 (0.089)	0.063 (0.090)	0.068 (0.105)	S
				0.063 (0.092)	0.063 (0.092)	0.063 (0.090)	0.063 (0.091)	0.063 (0.090)	0.063 (0.091)	0.068 (0.105)	Bi
				0.062 (0.089)	0.062 (0.088)	0.062 (0.088)	0.062 (0.088)	0.062 (0.088)	0.062 (0.089)	0.067 (0.102)	G
				0.063 (0.090)	0.063 (0.092)	0.063 (0.092)	0.063 (0.092)	0.063 (0.089)	0.063 (0.091)	0.067 (0.099)	GPU
Rpim	0.032 (0.044)	0.025 (0.042)	0.016 (0.036)	0.016 (0.036)	0.016 (0.036)	0.016 (0.037)	0.016 (0.036)	0.016 (0.036)	0.016 (0.036)	0.017 (0.042)	S
				0.016 (0.037)	0.016 (0.037)	0.016 (0.036)	0.016 (0.036)	0.016 (0.036)	0.016 (0.037)	0.017 (0.043)	Bi
				0.016 (0.036)	0.016 (0.035)	0.016 (0.035)	0.016 (0.035)	0.016 (0.035)	0.016 (0.036)	0.017 (0.041)	G

				0.016 (0.036)	0.016 (0.037)	0.016 (0.037)	0.016 (0.037)	0.016 (0.036)	0.016 (0.036)	0.017 (0.040)	GPU
CC½	0.994 (0.991)	0.995 (0.990)	0.998 (0.993)	0.998 (0.993)	0.998 (0.993)	0.998 (0.992)	0.998 (0.993)	0.998 (0.993)	0.998 (0.993)	0.997 (0.988)	S
				0.998 (0.992)	0.998 (0.992)	0.998 (0.993)	0.998 (0.993)	0.998 (0.993)	0.998 (0.993)	0.997 (0.988)	Bi
				0.998 (0.993)	0.998 (0.993)	0.998 (0.993)	0.998 (0.993)	0.998 (0.993)	0.998 (0.993)	0.997 (0.989)	G
				0.998 (0.993)	0.998 (0.992)	0.998 (0.992)	0.998 (0.992)	0.998 (0.993)	0.998 (0.993)	0.997 (0.991)	GPU
Anomalous slope	1.381	1.72	4.142	4.135	4.124	4.137	4.117	4.14	4.145	3.746	S
				4.07	4.112	4.124	4.108	4.138	4.124	3.721	Bi
				4.21	4.196	4.212	4.197	4.202	4.216	3.781	G
				4.139	4.126	4.132	4.064	4.147	4.141	3.723	GPU
Reflections used for Rwork	7181	7181	7181	7181	7181	7181	7181	7181	7181	7181	S, Bi, G, GPU
Reflections used for Rfree	329	329	329	329	329	329	329	329	329	329	S, Bi, G, GPU
Rwork	0.1832	0.1794	0.1721	0.1721	0.1721	0.172	0.1721	0.1718	0.1722	0.1719	S
				0.1719	0.1719	0.1722	0.172	0.1722	0.1722	0.1718	Bi
				0.1719	0.1721	0.172	0.1721	0.172	0.1721	0.172	G
				0.1721	0.1719	0.172	0.172	0.1721	0.1722	0.1722	GPU
Rfree	0.2226	0.2207	0.2183	0.2184	0.2181	0.2182	0.2183	0.2181	0.2183	0.2178	S
				0.2183	0.218	0.2181	0.2177	0.2184	0.2186	0.2183	Bi
				0.2184	0.2177	0.2183	0.2185	0.2183	0.2181	0.2178	G
				0.2182	0.2183	0.218	0.2181	0.2185	0.2182	0.2177	GPU
CCwork	0.9335	0.9343	0.942	0.9422	0.9422	0.9422	0.942	0.9422	0.942	0.9426	S
				0.9422	0.9422	0.9421	0.9422	0.9421	0.942	0.9426	Bi
				0.9422	0.9421	0.9421	0.9421	0.9421	0.942	0.9425	G
				0.9421	0.9422	0.9422	0.9422	0.9421	0.942	0.9424	GPU
CCfree	0.8817	0.8808	0.8888	0.889	0.8892	0.8892	0.8887	0.8898	0.8887	0.8905	S
				0.8892	0.8894	0.8889	0.8898	0.8887	0.8889	0.8903	Bi
				0.8889	0.8891	0.8889	0.8888	0.8889	0.8888	0.8905	G
				0.8888	0.889	0.8893	0.8893	0.8887	0.889	0.8904	GPU
Rms BondLength	0.0098	0.0099	0.0095	0.0095	0.0095	0.0095	0.0095	0.0096	0.0095	0.0096	S
				0.0095	0.0095	0.0095	0.0095	0.0095	0.0094	0.0096	Bi
				0.0095	0.0095	0.0095	0.0095	0.0095	0.0095	0.0095	G
				0.0095	0.0095	0.0095	0.0095	0.0095	0.0095	0.0096	GPU
Rms BondAngle	1.7533	1.7547	1.7495	1.7522	1.7516	1.7471	1.7495	1.7625	1.7518	1.7501	S
				1.7469	1.7473	1.7502	1.7509	1.7519	1.7501	1.7489	Bi
				1.7500	1.7473	1.7455	1.7497	1.7457	1.7458	1.7434	G
				1.7494	1.7482	1.7469	1.7462	1.7527	1.7506	1.7494	GPU

S2 Anomalous peak heights

This section includes the tables of detailed anomalous peak heights ($> 5\sigma$) of three test crystals after applying sampling methods (S7, S9, and S11) and acceleration methods (S8, S10, and S12), Insulin, Thermolysin and Thaumatin. The abbreviations in the method column in S7, S9, and S11 are Sy: Systematic sampling; R: Random sampling; RS: Randomised Systematic sampling; St: Stratified sampling. The abbreviations in the method column in S8, S10, and S12 are S: Standard method; Bi: Bisection method; G: Gridding method; GPU: CUDA implementation. For those blank cells in the column of No, it can't identify the peak heights of those atoms.

Table S7: Anomalous peak heights of Insulin of different sampling methods ($> 5\sigma$)

Nearest atom	No	SH	ACSH	ACSH	ACSH	ACSH	ACSH	ACSH	ACSH	ACSH	Method
Ratio (%)			100	1	0.5	0.1	0.05	0.01	0.005	0.001	
SG_B:CYS19	14.61	17.59	17.39	17.38	17.38	17.38	17.38	17.41	17.37	17.27	Sy
SG_B:CYS19				17.38	17.38	17.42	17.42	17.4	17.36	17.17	R
SG_B:CYS19				17.4	17.38	17.37	17.38	17.37	17.38	17.37	RS
SG_B:CYS19				17.39	17.38	17.39	17.37	17.36	17.38	17.3	St
SG_A:CYS6	12.28	13.92	14.18	14	14	14	14	14.18	14.01	13.96	Sy
SG_A:CYS6				13.99	14	13.99	13.99	14.19	14.12	13.88	R
SG_A:CYS6				14.18	14	14	13.99	13.97	13.97	14	RS
SG_A:CYS6				14.18	14	14.18	13.99	14	14.17	14.12	St
SG_A:CYS20	12.03	13.61	13.86	13.7	13.7	13.7	13.7	13.85	13.68	13.76	Sy
SG_A:CYS20				13.7	13.7	13.7	13.7	13.85	13.83	13.68	R
SG_A:CYS20				13.85	13.7	13.7	13.71	13.72	13.71	13.69	RS
SG_A:CYS20				13.85	13.7	13.86	13.71	13.71	13.88	13.93	St
SG_A:CYS11	11.85	13.27	13.44	13.38	13.38	13.38	13.37	13.44	13.39	13.43	Sy
SG_A:CYS11				13.38	13.38	13.33	13.32	13.36	13.41	13.43	R
SG_A:CYS11				13.44	13.38	13.38	13.38	13.37	13.37	13.47	RS
SG_A:CYS11				13.44	13.38	13.44	13.38	13.39	13.44	13.45	St
SG_B:CYS7	8.98	11.16	11.08	11.15	11.15	11.15	11.15	11.08	11.13	11.2	Sy
SG_B:CYS7				11.15	11.15	11.16	11.16	11.09	11.1	11.15	R
SG_B:CYS7				11.08	11.15	11.15	11.16	11.23	11.19	11.21	RS
SG_B:CYS7				11.08	11.15	11.08	11.16	11.19	11.15	11.19	St
SG_A:CYS7	8.25	10.23	10.44	10.3	10.3	10.3	10.39	10.44	10.3	10.28	Sy
SG_A:CYS7				10.3	10.3	10.33	10.33	10.45	10.43	10.31	R
SG_A:CYS7				10.44	10.3	10.3	10.3	10.39	10.39	10.34	RS
SG_A:CYS7				10.44	10.3	10.44	10.29	10.38	10.43	10.37	St

Table S8: Anomalous peak heights of Insulin of different acceleration methods ($> 5\sigma$)

Nearest atom	No	SH	ACSH	ACSH	ACSH	ACSH	ACSH	ACSH	ACSH	ACSH	Method
Ratio (%)			100	1	0.5	0.1	0.05	0.01	0.005	0.001	
SG_B:CYS19	14.61	17.59	17.39	17.38	17.38	17.38	17.38	17.41	17.37	17.27	S
SG_B:CYS19				17.4	17.38	17.4	17.4	17.39	17.38	17.26	Bi
SG_B:CYS19				17.38	17.38	17.38	17.43	17.4	17.39	17.26	G
SG_B:CYS19				17.4	17.38	17.4	17.38	17.41	17.38	17.27	GPU
SG_A:CYS6	12.28	13.92	14.18	14	14	14	14	14.18	14.01	13.96	S

SG_A:CYS6				14.18	14	14.18	14.18	14.18	14.01	13.9	Bi
SG_A:CYS6				14.18	14.18	14.18	14.17	14.18	14.19	13.89	G
SG_A:CYS6				14.18	14	14.18	14	14.18	14.19	13.89	GPU
SG_A:CYS20	12.03	13.61	13.86	13.7	13.7	13.7	13.7	13.85	13.68	13.76	S
SG_A:CYS20				13.86	13.7	13.86	13.86	13.85	13.67	13.75	Bi
SG_A:CYS20				13.87	13.87	13.87	13.88	13.87	13.83	13.77	G
SG_A:CYS20				13.85	13.7	13.85	13.7	13.85	13.83	13.76	GPU
SG_A:CYS11	11.85	13.27	13.44	13.38	13.38	13.38	13.37	13.44	13.38	13.43	S
SG_A:CYS11				13.36	13.38	13.43	13.43	13.44	13.39	13.44	Bi
SG_A:CYS11				13.45	13.45	13.44	13.4	13.38	13.46	13.43	G
SG_A:CYS11				13.44	13.38	13.44	13.37	13.44	13.45	13.44	GPU
SG_B:CYS7	8.98	11.16	11.08	11.15	11.15	11.15	11.15	11.08	11.13	11.2	S
SG_B:CYS7				11.08	11.15	11.08	11.08	11.07	11.14	11.23	Bi
SG_B:CYS7				11.1	11.1	11.1	11.11	11.1	11.08	11.23	G
SG_B:CYS7				11.08	11.15	11.08	11.15	11.08	11.06	11.23	GPU
SG_A:CYS7	8.25	10.23	10.44	10.3	10.3	10.3	10.3	10.44	10.3	10.28	S
SG_A:CYS7				10.45	10.3	10.45	10.45	10.45	10.3	10.32	Bi
SG_A:CYS7				10.45	10.45	10.45	10.46	10.45	10.46	10.32	G
SG_A:CYS7				10.44	10.3	10.44	10.3	10.44	10.45	10.32	GPU

Table S9: Anomalous peak heights of Thermolysin of different sampling methods ($> 5\sigma$)

Nearest atom	No	SH	ACSH	ACSH	ACSH	ACSH	ACSH	ACSH	ACSH	ACSH	Method
Ratio (%)			100	1	0.5	0.1	0.05	0.01	0.005	0.001	
SD_A:MET205	12.52	18.88	21.54	21.45	21.47	21.46	21.54	21.43	21.4	21.49	Sy
SD_A:MET205				21.4	21.45	21.48	21.48	21.39	21.42	21.38	R
SD_A:MET205				21.49	21.51	21.45	21.55	21.45	21.44	21.51	RS
SD_A:MET205				21.51	21.62	21.49	21.56	21.59	21.4	21.57	St
ZN_A:ZN405	9.77	15	16.12	16.05	16.12	15.96	16.06	16.07	16.3	16.18	Sy
ZN_A:ZN405				15.93	16.05	16.08	16.06	16.02	16.06	16.06	R
ZN_A:ZN405				16.07	16.12	16.12	16.15	16.11	16.15	16.02	RS
ZN_A:ZN405				16.16	16.16	15.96	16.14	16.15	15.93	16.25	St
SD_A:MET120	7.69	11.97	12.7	12.58	12.59	12.7	12.64	12.63	12.75	12.53	Sy
SD_A:MET120				12.64	12.58	12.64	12.62	12.73	12.72	12.69	R
SD_A:MET120				12.7	12.59	12.59	12.59	12.64	12.66	12.55	RS
SD_A:MET120				12.57	12.69	12.69	12.64	12.65	12.69	12.57	St
S_A:SO41003	8.3	11.29	11.72	11.72	11.71	11.79	11.75	11.69	11.59	11.71	Sy
S_A:SO41003				11.68	11.74	11.6	11.6	11.7	11.98	11.99	R
S_A:SO41003				11.59	11.7	11.69	11.75	11.63	11.78	11.89	RS
S_A:SO41003				11.68	11.77	11.78	11.69	11.78	11.62	11.55	St
S_A:SO41006	6.74	7.26	7.84	7.81	7.85	7.84	7.78	7.78	7.87	7.63	Sy
S_A:SO41006				7.83	7.81	7.77	7.86	7.76	7.69	7.71	R
S_A:SO41006				7.8	7.83	7.82	7.88	7.83	7.62	7.92	RS
S_A:SO41006				7.81	7.83	7.75	7.83	7.81	7.81	7.66	St
CL_A:CL406	4.65	6.05	6.75	6.75	6.76	6.77	6.78	6.75	6.91	6.61	Sy
CL_A:CL406				6.77	6.75	6.76	6.74	6.82	6.98	6.79	R
CL_A:CL406				6.79	6.73	6.75	6.74	6.77	6.74	6.56	RS

CL_A:CL406				6.71	6.75	6.79	6.73	6.72	6.82	6.73	St
S_A:SO41004	4.31	5.72	6.4	6.37	6.38	6.37	6.37	6.43	6.28	6.51	Sy
S_A:SO41004				6.44	6.38	6.41	6.39	6.34	6.44	6.66	R
S_A:SO41004				6.39	6.4	6.37	6.34	6.26	6.67	6.19	RS
S_A:SO41004				6.37	6.33	6.43	6.33	6.32	6.35	6.49	St
S_A:SO41005		6	6.34	6.41	6.4	6.35	6.39	6.42	6.26	6.27	Sy
S_A:SO41005				6.37	6.42	6.35	6.39	6.24	6.31	6.1	R
S_A:SO41005				6.34	6.35	6.4	6.41	6.38	6.28	6.34	RS
S_A:SO41005				6.38	6.35	6.3	6.38	6.38	6.38	6.32	St
S_A:SO41007	4.92	6.75	6.11	6.11	6.1	6.09	6.12	6.1	6.4	6.09	Sy
S_A:SO41007				6.09	6.09	6.07	6.11	6.11	6.3	6.01	R
S_A:SO41007				6.09	6.11	6.09	6.19	6.15	5.99	6.12	RS
S_A:SO41007				6.2	6.18	6.12	6.24	6.16	6.11	6.02	St
S_A:SO41008		5.15	5.44	5.45	5.41	5.49	5.46	5.44	5.59	5.35	Sy
S_A:SO41008				5.47	5.44	5.47	5.5	5.6	5.63	5.2	R
S_A:SO41008				5.48	5.45	5.43	5.52	5.65	5.39	5.31	RS
S_A:SO41008				5.54	5.48	5.43	5.56	5.5	5.6	5.3	St

Table S10: Anomalous peak heights of Thermolysin of different acceleration methods ($> 5\sigma$)

Nearest atom	No	SH	ACSH	ACSH	ACSH	ACSH	ACSH	ACSH	ACSH	ACSH	Method
Ratio (%)			100	1	0.5	0.1	0.05	0.01	0.005	0.001	
SD_A:MET205	12.52	18.88	21.54	21.45	21.47	21.46	21.54	21.43	21.4	21.49	S
SD_A:MET205				21.36	21.43	21.43	21.39	21.4	21.67	21.54	Bi
SD_A:MET205				21.45	21.44	21.47	21.45	21.52	21.49	21.52	G
SD_A:MET205				21.45	21.5	21.41	21.4	21.39	21.43	21.49	GPU
ZN_A:ZN405	9.77	15	16.12	16.05	16.12	15.96	16.06	16.07	16.3	16.18	S
ZN_A:ZN405				15.93	15.93	15.95	15.99	15.92	16.14	16.06	Bi
ZN_A:ZN405				16.24	16.29	16.15	16.17	16.1	16.29	16.07	G
ZN_A:ZN405				16.04	16.11	15.93	16.08	15.94	16.28	16.09	GPU
SD_A:MET120	7.69	11.97	12.7	12.58	12.59	12.7	12.64	12.63	12.75	12.53	S
SD_A:MET120				12.69	12.72	12.7	12.69	12.65	12.67	12.66	Bi
SD_A:MET120				12.67	12.67	12.59	12.61	12.63	12.6	12.56	G
SD_A:MET120				12.62	12.68	12.65	12.74	12.66	12.77	12.6	GPU
S_A:SO41003	8.3	11.29	11.72	11.72	11.71	11.79	11.75	11.69	11.59	11.71	S
S_A:SO41003				11.74	11.69	11.73	11.8	11.78	11.74	11.69	Bi
S_A:SO41003				11.68	11.58	11.56	11.65	11.61	11.49	11.58	G
S_A:SO41003				11.68	11.7	11.72	11.73	11.72	11.6	11.62	GPU
S_A:SO41006	6.74	7.26	7.84	7.81	7.85	7.84	7.78	7.78	7.87	7.63	S
S_A:SO41006				7.78	7.82	7.74	7.78	7.75	7.78	7.67	Bi
S_A:SO41006				7.83	7.81	7.84	7.82	7.82	7.75	7.86	G
S_A:SO41006				7.82	7.87	7.78	7.69	7.82	7.83	7.58	GPU
CL_A:CL406	4.65	6.05	6.75	6.75	6.76	6.77	6.78	6.75	6.91	6.61	S
CL_A:CL406				6.78	6.71	6.75	6.78	6.7	6.77	6.55	Bi
CL_A:CL406				6.78	6.84	6.76	6.75	6.73	6.87	6.77	G
CL_A:CL406				6.75	6.75	6.8	6.86	6.76	6.94	6.59	GPU
S_A:SO41004	4.31	5.72	6.4	6.37	6.38	6.37	6.37	6.43	6.28	6.51	S

S_A:SO41004				6.32	6.37	6.39	6.37	6.4	6.26	6.48	Bi
S_A:SO41004				6.36	6.38	6.45	6.44	6.45	6.35	6.43	G
S_A:SO41004				6.38	6.37	6.45	6.38	6.43	6.24	6.53	GPU
S_A:SO41005		6	6.34	6.41	6.4	6.35	6.39	6.42	6.26	6.27	S
S_A:SO41005				6.34	6.42	6.41	6.33	6.35	6.34	6.32	Bi
S_A:SO41005				6.41	6.37	6.49	6.4	6.49	6.26	6.47	G
S_A:SO41005				6.44	6.39	6.37	6.33	6.42	6.28	6.27	GPU
S_A:SO41007	4.92	6.75	6.11	6.11	6.1	6.09	6.12	6.1	6.4	6.09	S
S_A:SO41007				6.08	6.05	6.09	6.15	6.1	6.18	6.06	Bi
S_A:SO41007				6.17	6.18	6.09	6.14	6.03	6.29	6.11	G
S_A:SO41007				6.13	6.13	6.08	5.98	6.1	6.37	5.99	GPU
S_A:SO41008		5.15	5.44	5.45	5.41	5.49	5.46	5.44	5.59	5.35	S
S_A:SO41008				5.59	5.55	5.54	5.55	5.55	5.6	5.52	Bi
S_A:SO41008				5.6	5.58	5.51	5.49	5.53	5.54	5.51	G
S_A:SO41008				5.47	5.44	5.45	5.48	5.48	5.68	5.41	GPU

Table S11: Anomalous peak heights of Thaumatin of different sampling methods ($> 5\sigma$)

Nearest atom	No	SH	ACSH	ACSH	ACSH	ACSH	ACSH	ACSH	ACSH	ACSH	Method
Ratio (%)			100	1	0.5	0.1	0.05	0.01	0.005	0.001	
SG_A:CYS56	16.35	16.48	16.61	16.6	16.59	16.65	16.61	16.66	16.63	16.28	Sy
SG_A:CYS56				16.59	16.63	16.6	16.58	16.57	16.64	16.65	R
SG_A:CYS56				16.61	16.62	16.69	16.66	16.61	16.63	16.61	RS
SG_A:CYS56				16.64	16.6	16.66	16.65	16.65	16.67	16.63	St
SG_A:CYS9	15.01	15.26	16.07	16.14	16.14	16.04	16.07	16.14	16.16	15.85	Sy
SG_A:CYS9				16.14	16.05	16.06	16.13	16.12	16.1	16.03	R
SG_A:CYS9				16.07	16.15	16.03	16.05	16.15	16.06	16.18	RS
SG_A:CYS9				16.15	16.07	16.01	16.01	16.09	16.17	16.16	St
SG_A:CYS145	13.74	14.55	15.64	15.76	15.76	15.66	15.65	15.71	15.72	15.26	Sy
SG_A:CYS145				15.76	15.67	15.64	15.75	15.73	15.66	15.64	R
SG_A:CYS145				15.64	15.76	15.67	15.65	15.22	15.63	15.76	RS
SG_A:CYS145				15.77	15.65	15.66	15.67	15.64	15.75	15.65	St
SD_A:MET112	13.86	14.68	15.44	15.52	15.51	15.53	15.43	15.54	15.59	15.34	Sy
SD_A:MET112				15.52	15.53	15.45	15.53	15.51	15.44	15.38	R
SD_A:MET112				15.44	15.53	15.58	15.53	15.53	15.43	15.6	RS
SD_A:MET112				15.52	15.43	15.52	15.53	15.44	15.55	15.56	St
SG_A:CYS149	13.87	14.37	14.81	14.82	14.83	14.73	14.8	14.85	14.85	14.57	Sy
SG_A:CYS149				14.82	14.71	14.8	14.83	14.8	14.81	14.85	R
SG_A:CYS149				14.81	14.84	14.7	14.73	14.84	14.73	14.81	RS
SG_A:CYS149				14.83	14.8	14.7	14.71	14.8	14.86	14.87	St
SG_A:CYS77	13.32	13.89	14.73	14.65	14.64	14.68	14.68	14.62	14.59	14.4	Sy
SG_A:CYS77				14.65	14.7	14.73	14.64	14.68	14.68	14.67	R
SG_A:CYS77				14.72	14.64	14.67	14.68	14.63	14.72	14.55	RS
SG_A:CYS77				14.59	14.72	14.68	14.66	14.7	14.63	14.72	St
SG_A:CYS193	13.16	14.08	14.45	14.49	14.49	14.43	14.42	14.47	14.49	14.18	Sy
SG_A:CYS193				14.48	14.42	14.43	14.48	14.45	14.44	14.37	R
SG_A:CYS193				14.44	14.5	14.44	14.44	14.48	14.42	14.54	RS

SG_A:CYS193				14.48	14.44	14.43	14.44	14.47	14.53	14.48	St
SG_A:CYS204	12.45	12.71	13.17	13.2	13.2	13.09	13.18	13.2	13.2	13.1	Sy
SG_A:CYS204				13.2	13.08	13.19	13.19	13.17	13.17	13.16	R
SG_A:CYS204				13.18	13.2	13.13	13.09	13.22	13.16	13.27	RS
SG_A:CYS204				13.19	13.18	13.12	13.12	13.18	13.18	13.15	St
SG_A:CYS71	11.51	12	12.62	12.52	12.52	12.56	12.59	12.48	12.46	12.33	Sy
SG_A:CYS71				12.52	12.59	12.62	12.53	12.53	12.6	12.62	R
SG_A:CYS71				12.61	12.52	12.59	12.57	12.54	12.62	12.58	RS
SG_A:CYS71				12.49	12.61	12.59	12.56	12.59	12.49	12.59	St
SG_A:CYS164	12.18	12.16	12.4	12.45	12.44	12.43	12.4	12.42	12.46	12.34	Sy
SG_A:CYS164				12.44	12.42	12.41	12.45	12.42	12.39	12.4	R
SG_A:CYS164				12.42	12.44	12.38	12.43	12.46	12.42	12.47	RS
SG_A:CYS164				12.44	12.41	12.42	12.43	12.42	12.47	12.41	St
SG_A:CYS66	12.01	12.14	12.29	12.32	12.3	12.56	12.56	12.34	12.33	12.11	Sy
SG_A:CYS66				12.31	12.3	12.27	12.28	12.3	12.56	12.26	R
SG_A:CYS66				12.3	12.31	12.59	12.57	12.33	12.33	12.54	RS
SG_A:CYS66				12.29	12.29	12.55	12.58	12.32	12.32	12.28	St
SG_A:CYS121	11.97	12.15	12.29	12.24	12.23	12.26	12.28	12.22	12.25	12.13	Sy
SG_A:CYS121				12.23	12.29	12.29	12.25	12.26	12.29	12.32	R
SG_A:CYS121				12.29	12.25	12.28	12.28	12.27	12.34	12.25	RS
SG_A:CYS121				12.23	12.29	12.28	12.27	12.31	12.25	12.33	St
SG_A:CYS126	10.57	10.74	11.53	11.49	11.49	11.54	11.51	11.53	11.51	11.37	Sy
SG_A:CYS126				11.49	11.56	11.54	11.51	11.49	11.5	11.43	R
SG_A:CYS126				11.54	11.5	11.52	11.53	11.5	11.5	11.45	RS
SG_A:CYS126				11.49	11.54	11.57	11.54	11.57	11.51	11.55	St

Table S12: Anomalous peak heights of Thaumatin of different acceleration methods ($> 5\sigma$)

Nearest atom	No	SH	ACSH	ACSH	ACSH	ACSH	ACSH	ACSH	ACSH	ACSH	Method
Ratio (%)			100	1	0.5	0.1	0.05	0.01	0.005	0.001	
SG_A:CYS56	16.35	16.48	16.61	16.6	16.59	16.65	16.61	16.66	16.63	16.28	S
SG_A:CYS56				16.63	16.62	16.59	16.61	16.65	16.63	16.29	Bi
SG_A:CYS56				16.64	16.63	16.65	16.61	16.65	16.61	16.33	G
SG_A:CYS56				16.61	16.63	16.64	16.62	16.64	16.58	16.45	GPU
SG_A:CYS9	15.01	15.26	16.07	16.14	16.14	16.04	16.07	16.14	16.16	15.85	S
SG_A:CYS9				16.03	16.03	16.14	16.17	16.14	16.15	15.83	Bi
SG_A:CYS9				16.07	16.08	16.09	16.06	16.08	16.1	15.79	G
SG_A:CYS9				16.07	16.03	16.03	15.98	16.14	16.14	15.86	GPU
SG_A:CYS145	13.74	14.55	15.64	15.76	15.76	15.66	15.65	15.71	15.72	15.26	S
SG_A:CYS145				15.67	15.65	15.76	15.76	15.72	15.7	15.26	Bi
SG_A:CYS145				15.62	15.63	15.64	15.62	15.61	15.58	15.15	G
SG_A:CYS145				15.65	15.67	15.67	15.7	15.74	15.72	15.24	GPU
SD_A:MET112	13.86	14.68	15.44	15.52	15.51	15.53	15.43	15.54	15.59	15.34	S
SD_A:MET112				15.52	15.52	15.52	15.52	15.52	15.62	15.39	Bi
SD_A:MET112				15.44	15.44	15.44	15.42	15.44	15.48	15.31	G
SD_A:MET112				15.44	15.52	15.54	15.47	15.51	15.56	15.35	GPU
SG_A:CYS149	13.87	14.37	14.81	14.82	14.83	14.73	14.8	14.85	14.85	14.57	S

SG_A:CYS149				14.69	14.7	14.82	14.81	14.84	14.83	14.59	Bi
SG_A:CYS149				14.78	14.75	14.75	14.79	14.78	14.82	14.49	G
SG_A:CYS149				14.8	14.71	14.72	14.68	14.84	14.83	14.53	GPU
SG_A:CYS77	13.32	13.89	14.73	14.65	14.64	14.68	14.68	14.62	14.59	14.4	S
SG_A:CYS77				14.69	14.69	14.64	14.59	14.58	14.62	14.33	Bi
SG_A:CYS77				14.7	14.68	14.7	14.69	14.68	14.71	14.48	G
SG_A:CYS77				14.73	14.7	14.68	14.68	14.58	14.65	14.48	GPU
SG_A:CYS193	13.16	14.08	14.45	14.49	14.49	14.43	14.42	14.47	14.49	14.18	S
SG_A:CYS193				14.42	14.42	14.48	14.5	14.47	14.52	14.2	Bi
SG_A:CYS193				14.46	14.46	14.46	14.43	14.48	14.45	14.15	G
SG_A:CYS193				14.44	14.41	14.44	14.4	14.47	14.5	14.26	GPU
SG_A:CYS204	12.45	12.71	13.17	13.2	13.2	13.09	13.18	13.2	13.2	13.1	S
SG_A:CYS204				13.09	13.05	13.19	13.19	13.19	13.2	13.11	Bi
SG_A:CYS204				13.16	13.17	13.16	13.18	13.16	13.23	13.09	G
SG_A:CYS204				13.17	13.07	13.08	13.09	13.21	13.21	13.12	GPU
SG_A:CYS71	11.51	12	12.62	12.52	12.52	12.56	12.59	12.48	12.46	12.33	S
SG_A:CYS71				12.59	12.58	12.5	12.5	12.48	12.47	12.31	Bi
SG_A:CYS71				12.64	12.63	12.64	12.63	12.61	12.63	12.47	G
SG_A:CYS71				12.61	12.59	12.57	12.59	12.48	12.49	12.41	GPU
SG_A:CYS164	12.18	12.16	12.4	12.45	12.44	12.43	12.4	12.42	12.46	12.34	S
SG_A:CYS164				12.41	12.42	12.44	12.47	12.45	12.5	12.29	Bi
SG_A:CYS164				12.39	12.38	12.39	12.36	12.39	12.36	12.24	G
SG_A:CYS164				12.41	12.43	12.43	12.44	12.44	12.45	12.31	GPU
SG_A:CYS66	12.01	12.14	12.29	12.32	12.3	12.56	12.56	12.34	12.33	12.11	S
SG_A:CYS66				12.3	12.3	12.31	12.35	12.28	12.37	12.14	Bi
SG_A:CYS66				12.32	12.32	12.62	12.35	12.33	12.59	12.14	G
SG_A:CYS66				12.3	12.3	12.57	12.55	12.3	12.33	12.17	GPU
SG_A:CYS121	11.97	12.15	12.29	12.24	12.23	12.26	12.28	12.22	12.25	12.13	S
SG_A:CYS121				12.29	12.29	12.23	12.21	12.24	12.24	12.15	Bi
SG_A:CYS121				12.34	12.34	12.34	12.34	12.32	12.36	12.21	G
SG_A:CYS121				12.29	12.28	12.28	12.26	12.21	12.23	12.26	GPU
SG_A:CYS126	10.57	10.74	11.53	11.49	11.49	11.54	11.51	11.53	11.51	11.37	S
SG_A:CYS126				11.58	11.54	11.49	11.45	11.52	11.51	11.34	Bi
SG_A:CYS126				11.54	11.51	11.52	11.47	11.54	11.58	11.35	G
SG_A:CYS126				11.54	11.53	11.52	11.53	11.5	11.49	11.35	GPU

S3 3D rendering of tomography reconstruction

References

[1] Beilsten-Edmands, J., Winter, G., Gildea, R., Parkhurst, J., Waterman, D., and Evans, G. (2020). Scaling diffraction data in the *DIALS* software package: algorithms and new approaches for multi-crystal scaling. *Acta Cryst.*, D76:385–399.

[2] Lu, Y., Duman, R., Beilsten-Edmands, J., Winter, G., Basham, M., Evans, G., Kamps, J. J. A. G., Orville, A. M., Kwong, H.-S., Beis, K., Armour, W., and Wagner, A. (2024). Ray-tracing analytical absorption correction for X-ray crystallography based on tomographic reconstructions. *J. Appl. Cryst.*, 57(3).
